# B Cell Receptor Signaling and Protein Kinase D2 Support Regulatory B Cell Function in Pancreatic Cancer

**DOI:** 10.3389/fimmu.2021.745873

**Published:** 2022-01-03

**Authors:** Daniel Michaud, Bhalchandra Mirlekar, Colleen Steward, Gail Bishop, Yuliya Pylayeva-Gupta

**Affiliations:** ^1^ Department of Cell Biology and Physiology, The University of North Carolina at Chapel Hill School of Medicine, Chapel Hill, NC, United States; ^2^ Lineberger Comprehensive Cancer Center, The University of North Carolina at Chapel Hill School of Medicine, Chapel Hill, NC, United States; ^3^ Department of Microbiology and Immunology, The University of North Carolina at Chapel Hill School of Medicine, Chapel Hill, NC, United States; ^4^ Department of Microbiology and Immunology, The University of Iowa, Iowa City, IA, United States; ^5^ Holden Comprehensive Cancer Center, The University of Iowa, Iowa City, IA, United States; ^6^ Department of Genetics, The University of North Carolina at Chapel Hill School of Medicine, Chapel Hill, NC, United States

**Keywords:** regulatory B cell, IL-35, pancreatic cancer, PKD, BCR

## Abstract

B cells can act as potent suppressors of anti-tumor T cell immunity, presenting a mechanism of resistance to immunotherapy. In pancreatic ductal adenocarcinoma, B cells can display a T cell-suppressive or regulatory phenotype centered on the expression of the cytokine Interleukin 35 (IL-35). While B cell-mediated immunosuppression presents a barrier to anti-tumorigenic T cell function, it is not clear how regulatory B cell function could be targeted, and the signals that promote this suppressive phenotype in B cells are not well understood. Here we use a novel IL-35 reporter model to understand which signaling pathways are important for immunosuppressive properties in B cells. *In vitro* analysis of IL-35 reporter B cells revealed a synergy between the BCR and TLR4 signaling pathways is sufficient to induce IL-35 expression. However, *in vivo*, B cell receptor activation, as opposed to MyD88 signaling in B cells, is central to B cell-mediated suppression and promotion of pancreatic cancer growth. Further analysis identified protein kinase D2 (PKD2) as being a key downstream regulator of IL-35 expression in B cells. Regulatory B cells with an inactivating mutation in PKD2 failed to produce IL-35 or fully suppress effector T cell function *in vitro*. Furthermore, inhibition of PKD in B cells decreased tumor growth and promoted effector T cell function upon adoptive transfer into B cell-deficient mice. Collectively, these data provide insight into how regulatory B cell function is promoted in pancreatic cancer and identify potential therapeutic targets to restrain this function.

## Introduction

Pancreatic ductal adenocarcinoma (PDAC) is an aggressive malignancy with few viable treatment options for late-stage tumors resulting in a 10% 5-year survival rate (1). Pancreatic tumorigenesis is fueled by a robust infiltration of immunosuppressive cells such as regulatory T cells, myeloid-derived suppressor cells, and M2 macrophages (2–4). We previously identified B cells as key suppressors of T cell infiltration and effector function within pancreatic tumors, predominantly through production of the cytokine IL-35 (5, 6). However, the key regulators of this suppressive activity by B cells in the tumor microenvironment are still unclear.

Immunosuppressive or ‘regulatory’ B cells were originally identified in many disease contexts outside of cancer. The contribution of these B cell populations on disease regulation has been primarily linked to expression of anti-inflammatory IL-10 by to suppress host T cell responses (7–10). We and others have shown that B cells can also suppress T-cell activity in autoimmunity, infection, and cancer *via* the cytokine IL-35 (5, 6, 11–13). PDAC progression specifically, is dependent on IL-35 and not IL-10 production by B cells (5, 13). IL-35, a heterodimeric cytokine from the IL-12 family of cytokines, consists of p35 (*Il12a*) and EBi3 (*Ebi3*) subunits that potently suppress T cell immunity (14). Expression of the IL-35 subunits and IL-10 by B cells has been linked to stimulation of the B cell receptor (BCR) (8, 15), Toll-like receptors (TLRs) (10, 12, 16), and/or CD40 (12, 17). However, it is difficult to dissect precise expression pattern of the IL-35 subunits in the same B cell, due to the cytokine’s heterodimeric nature. To enable studies on regulation of IL-35 expression in B cells, we generated an *Il12a*-GFP reporter *mouse* (18) to accompany a previously generated *Ebi3*-TdTomato reporter mouse (19).

The exact antigen receptor signals acting on B cells to elicit immunosuppression have not been identified, but plausible BCR and/or TLR specific antigens are present within the pancreatic tumor microenvironment (20–24). These signals come in the forms of tumor-associated antigens, damage-associated molecular patterns, and a tumor-associated pancreatic microbiota (25–27). The roles of the BCR and TLRs on regulatory B cell activity in PDAC and ultimately promotion of pancreatic tumor growth have not been thoroughly examined. This study addresses the respective roles of BCR and TLR4 signaling by B cells in pancreatic cancer and how they regulate expression of IL-35.

## Materials and Methods

### Mouse Models

All mouse protocols were reviewed and approved by the Institutional Animal Care and Use Committee of the University of North Carolina at Chapel Hill (Chapel Hill, NC, USA). Animals were maintained in a specific pathogen-free facility. Six- to 8-week-old wild-type (WT) *C57Bl/6J* mice were purchased from The Charles River Laboratories (strain #027). 6- to 8-week-old *MD4* (strain #002595), *µMT* (strain #002288), *Prkd2S707A/S711A* (strain #017285), *CD19^Cre/+^* (strain #006785), and *Myd88^Fl/Fl^* (strain #008888) mice on C57Bl/6J background were purchased from Jackson Laboratories. *Ebi3^Tom/Tom^* mice were obtained from D. A. Vignali (University of Pittsburgh, Pittsburgh, PA) (19). *Il12a^GFP/GFP^* mice were generated at the University of North Carolina at Chapel Hill (Chapel Hill, NC, USA) (18). *Ebi3^Tom/Tom^; Il12a^GFP/GFP^* mice were generated by breeding *Ebi3^Tom/Tom^* mice with *Il12a^GFP/GFP^* mice until progeny were homozygous for both reporter alleles. *CD19^Cre/+^; Myd88^Fl/Fl^* mice were generated by breeding *CD19^Cre/+^* mice with *Myd88^Fl/Fl^* mice until progeny were homozygous for the floxed *Myd88* allele. *CD19^+/+^; Myd88^Fl/Fl^* littermates lacking Cre expression were used as controls.

### Cell Lines

The murine PDAC cell line KPC4662 (*p48* ^Cre/+^; LSL-*Kras* ^G12D/+^; LSL-*Tp53* ^R173/+^)was derived from a primary pancreatic tumor of C57Bl/6J KPC mice by Dr. Robert Vonderheide’s laboratory (University of Pennsylvania Perelman School of Medicine, Philadelphia, PA.) (28). Cells were maintained at 37°C and 5% CO2 in DMEM (#11995-065, Gibco) containing 10% Fetal calf serum (Corning) and 1x penicillin– streptomycin (#15140-122, Gibco). Cells were confirmed to be Mycoplasma and endotoxin free. KPC4662 Cells that were injected orthotopically were at <16 passages from original derivation.

### Orthotopic Tumor Modelling

For intrapancreatic injection of KPC4662 cancer cells, mice were anesthetized using a ketamine (100 mg/kg; Med-Vet International) and xylazine (10 mg/kg; Med-Vet International) cocktail. The depth of anesthesia was confirmed by verifying an absence of response to toe pinch. An incision in the left flank was made, and 1 x 10^5^ KPC4662 cells in ice-cold PBS mixed at 1:1 dilution with Matrigel (#354234, Corning) in a volume of 50 µL were injected using a 28-gauge needle into the tail of the pancreas. The wound was closed in two layers, with running 5-0 Vicryl RAPIDE sutures (Ethicon) for the body wall, and 5-0 PROLENE sutures (Ethicon) for the skin. All mice were given buprenorphine (0.1 mg/kg; Med-Vet International) subcutaneously after orthotopic surgery for pain relief. Tumors were grown in mice for 21 days unless otherwise noted. Tumor volume was measured using electronic calipers after animal sacrifice and calculated using the formula: Volume = Length * (Width^2^)/0.52.

### Depletion of CD4^+^ T Cells and CD8^+^ T Cells *In Vivo*

For CD4^+^ and CD8^+^ T-cell depletion studies, 200 µg of αCD4 (#BP0003-1, clone GK1.4, Bio X Cell) and 200 µg of αCD8 (#BP0004-1, clone 53-6.7, Bio X Cell) or an IgG isotype control rat IgG2b (#BE0086, clone MPC-11, Bio X Cell) and rat IgG2a (#BE0089, clone 2A3, Bio X Cell), respectively, were administered intraperitoneally daily starting 3 days prior to tumor cell injection and twice a week after tumor cell injection. Mice were sacrificed 21 days after tumor implantation. Depletion of T cells was confirmed by flow cytometry at time of animal sacrifice.

### Ultrasound Imaging of Orthotopic Tumors

Monitoring of orthotopic pancreatic tumors was performed weekly on the Vevo 2100 Imaging System (VisualSonics Inc.) using the MS550D transducer. Mice were anaesthetized using isoflurane (2%) throughout the procedure. Hair was removed from left flank of each mouse by electric razor and hair removal cream (Nair, Church & Dwight). Images were taken at 11 mm image depth and measurements were calculated using the Vevo LAB software (VisualSonics Inc.).

### Lymphocyte Isolation

Single-cell suspensions were prepared from tumors, spleens, and draining lymph nodes isolated from orthotopic models. Spleens and draining lymph nodes were mechanically disrupted using the plunger end of a 5 mL syringe and resuspended in 2% FCS/PBS. Pelleted samples were then depleted of red blood cells using RBC lysis buffer (eBioscience; 00-4333-57) and resuspended in 2% FCS/PBS. For isolation of tumor-infiltrating lymphocytes, tumor tissue was thoroughly minced using sterile razor blades and digested with collagenase IV (1.25 mg/mL; #LS004188, Worthington), 0.1% soybean trypsin inhibitor (#T9128, Sigma), hyaluronidase (1 mg/mL; #LS002592, Worthington), and DNase I (100 mg/mL; #LS002007, Worthington) in complete DMEM for 30 minutes at 37°C. Cell suspensions were passed through a 70µm cell strainer (#352350, Falcon) and resuspended in RPMI 1640 media (Gibco). Leukocytes were isolated from processed tumor tissues by Ficoll-Paque PLUS (#17-1440-03, GE Healthcare) density gradient centrifugation and resuspended in 2% FCS/PBS. For FACS sorted cell populations, cells were stained with fluorophore-labeled antibodies for 30 minutes on ice in FACS buffer (2% FCS/PBS). After staining, cells were washed twice with FACS buffer and resuspended in cell sorting buffer (1% FCS/PBS). Lymphocyte populations were sorted using a BD FACS ARIA III sorter to isolate CD19^+^CD21^Hi^CD5^+^CD1d^Hi^ Breg cells, CD19^+^CD21^Lo^CD5^-^CD1d^Lo^ Bcon cells, CD4^+^ T cells, and CD8^+^ T cells. Cells were collected in complete RPMI media containing 10% FCS with 1x penicillin–streptomycin (#15140-122, Gibco) antibiotics and 1x 2-β-Mercaptoethanol. For assays using total B cells, B cells were isolated from tissues using the EasySep Mouse B cell Isolation Kit (#19854, StemCell Technologies) according to manufacturer’s instructions. For adoptive transfer experiments, 6- to 8-week-old *µMT* mice were intravenously reconstituted with 1 x 10^7^ purified DMSO or CRT0066101 (PKDi) treated WT splenic B cells 16 hours prior to orthotopic injection. B cells were purified by negative magnetic selection (#19854, StemCell Technologies) and purity was >95% by flow cytometry. Both male and female mice were used in all studies.

### B Cell and T Cell *In Vitro* Cell Culture

Unless otherwise noted, B cells were activated with αCD40 (1 µg/mL, clone HM40-3, BioLegend), αIgM, µ chain (10µg/mL, #115-006-075, Jackson Immuno Research) and/or LPS (2 µg/mL, #L4391-1MG, Sigma) in complete RPMI for 48 hours as previously described (5, 6). For CD8^+^ and CD4^+^ T-cell culture, 1 x 10^5^ T cells were cultured in a 96-well plate with plate bound αCD3 (1 µg/mL, #BE0001-1, Clone 145-2C11, Bio X Cell) and soluble αCD28 (2 µg/mL, #BE0015-1, Clone 37.51, Bio X Cell) in complete RPMI for 48 hours. For detection of cytokines in B cells, 1 x 10^5^ cells were cultured using conditions indicated in figure legends for 48 hours in complete RPMI medium followed by the addition of PMA (50 ng/mL; #P8139, Sigma), Ionomycin (200 ng/mL; #9995S, Cell Signaling Technologies), and Brefeldin A (1x, #420601, BioLegend) for the final 5 hours at 37°C. For detection of cytokines in T cells, 1x 10^5^ cells were cultured with αCD3 and αCD28 for 48 hours with 1x Brefeldin A being added for the final 5 hours of culture. For transwell co-culture assays, a total of 1 x 10^5^ CD19^+^CD21^Hi^CD5^+^CD1d^Hi^ B cells and 1 x 10^5^ CD8^+^ T cells or 1 x 10^5^ CD4^+^ T cells were co-cultured in 96-well Transwell plates (#3381; Corning) with B cells occupying the top chamber and CD4^+^ or CD8^+^ T cells in the bottom chamber for 48 hours. For experiments utilizing kinase inhibitors, splenic B cells were cultured for 45 minutes with the indicated inhibitor prior to 48-hour stimulation *in vitro*. The following inhibitors were used: Selleckchem - ERKi (SCH772984, #S7101), PI3Ki (Idelalisib, #S2226), BTKi (Ibrutinib, #S2680); Cell Signaling Technologies – NFATi (FK-506, #9974S); Sigma – LYNi (Dasatinib, CDS023389-25MG); NF-kBi (Compound A) was a gift from Dr. Albert Baldwin (29). Inhibitor concentration was determined by the lowest amount of inhibitor required to decrease target activation of at least 75% from DMSO treated condition. Inhibition was quantified using ImageStudio software (LI-COR).

### Immunophenotyping by Flow Cytometry

Isolated immune cells were washed and blocked with αCD16/CD32 (100ug/100,000 cells, #101320, BioLegend) for 5 minutes on ice and then stained with labeled antibodies against surface markers or cytokines on ice for 30 minutes in FACS buffer (2% FCS/PBS). Intracellular staining for Foxp3 was performed using a Foxp3/Transcription Factor Staining Kit (#00-5523, eBioscience) following surface marker staining and was used according to manufacturer’s instructions. The following monoclonal antibodies directed against mouse antigens were used for flow cytometry: BioLegend - CD45 (clone 30-F11), CD3 (17A2), CD4 (GK1.5), Foxp3 (FJK-16S), CD8 (53-6.7), NK1.1 (PK136), CD11c (N418), CD19 (6D5), CD5 (53-7.3), CD21/35 (7E9), IgD (11–26), IgM (RMM-1), CD23 (B3B4), CD138 (281-2); BD Biosciences - CD1d (clone 1B1), CD93 (AA4.1), CD11b (M1/70). Viability of cells was determined by staining with either Zombie NIR (#423105, BioLegend) or Zombie Red (#423113, BioLegend) viability dye. All samples were acquired on LSRII Fortessa (BD Bioscience) at UNC Flow Cytometry Core Facility and analyzed by FlowJo version 10.2 (TreeStar, Inc.).

### Detection of Intracellular Cytokines by Flow Cytometry

Cells were washed and blocked with αCD16/CD32 (100ug/100,000 cells, #101320, BioLegend) for 5 minutes on ice. Cells were then washed and stained with labeled antibodies against surface markers on ice for 30 minutes in FACS buffer (2% FCS/PBS). After surface staining, cells were fixed and permeabilized using the Cytofix/Cytoperm Kit (#554714, BD Biosciences) for 20 minutes at 4°C. Intracellular staining was performed using fluorophore-conjugated cytokine antibodies for 1 hour at 4°C. Cells were then washed twice and resuspended in cell staining buffer (#420201, BioLegend). The following antibodies directed against mouse antigens were used for flow cytometry: BioLegend - IL-10 (clone JES5-16E3), IFNy (clone XMG1.2), TNFα (clone MPX-XT22); eBioscience - p35 (clone 4D10p35); R&D Systems - EBi3 (clone 355022). All samples were acquired on LSRII Fortessa (BD Bioscience) at the UNC Flow Cytometry Core Facility and analyzed by FlowJo version 10.2 (TreeStar, Inc.).

### Western Immunoblotting

Cells were lysed using RIPA Buffer (#89901, Thermo Fisher) containing HALT phosphatase/protease inhibitors (#78440, Thermo Fisher) on ice and then centrifuged for 10 minutes at 13000rpm for 10 minutes to extract supernatant. Lysates were quantified using Pierce BCA Protein Assay (#23225, Thermo Fisher) according to manufacturer’s instructions. 40µg of total protein was boiled for 5 minutes at 95°C, mixed with 6x laemmli loading buffer, and then loaded and into polyacrylamide gels. Protein was transferred onto LF-PVDF membranes (#162-0264, Bio-Rad) followed by overnight primary antibody incubation. Primary antibody was probed with fluorescent secondary antibodies and detected using the LI-COR Odyssey imager (LI-COR). Primary and secondary antibodies were diluted in 5% BSA/TBS for blocking. The following primary antibodies were used for immunoblotting: Cell Signaling Technologies – pErk1/2 Thr202/Tyr204 (D13.14.4E), Erk1/2 (L34F12), Nfat1 (D43B1), pP65 Ser536 (93H1), P65 (L846), pAkt Ser473 (D9E), Akt (40D4), pSyk Tyr525/526 (C87C1), pSrc Tyr416 (D49G4), Src (L4A1), pBtk Tyr223 (D9T6H), Btk (D6T2C), pPKD/PKCμ Ser744/748 (#2054S), β-Actin (13E5). The following secondary antibodies were used for primary antibody detection: IRDye 800CW Goat anti-Rabbit IgG (#925-32211, LI-COR), IRDye 680RD Goat anti-Mouse IgG (#925-68070, LI-COR).

### Quantitative PCR Analysis of Gene Expression

RNA was prepared from splenic, tumoral, and draining lymph node B cells of *CD19^Cre/+^; Myd88^Fl/Fl^* and *CD19^+/+^; Myd88^Fl/Fl^* tumor-bearing mice using the RNeasy Micro Kit (#74004, Qiagen). cDNA was generated from 1µg of total RNA using Maxima First-Strand cDNA synthesis RT Kit (#K1672, Thermo Fisher). qPCR analysis was performed using Perfecta SYBR Green FastMix (95072-250, Quantabio) on the QuantStudio 6 instrument (Applied Biosystems). Results were normalized to the expression of Tbp as an internal control, and each sample was run in triplicate. Gene expression was determined by the ΔΔCt method (2–ΔΔCt). The following primers were used for PCR amplification: *Tbp* FWD 5’-AGAACAATCCAGACTAGCAGCA–3’, *Tbp* REV 5’-GGGAACTTCACATCACAGCTC-3’, *Il10* FWD 5’-GCTCTTACTGATGGCATGAG-3’, *Il10* REV 5’-CGCAGCTCTAGGAGCATGTG-3’, *Ebi3* FWD 5’- CTTACAGGCTCGGTGTGGC-3’, *Ebi3* REV 5’-GTGACATTTAGCATGTAGGGCA-3’, *Il12a* FWD 5’-CATCGATGAGCTGATGCAGT-3’ *Il12a* REV 5’-CAGATAGCCCATCACCCTGT-3’, *Prkd1* FWD 5’-GGGGGCATCTCGTTCCATC-3’, *Prkd1* REV5’- GTGCCGAAAAAGCAGGATCTT, *Prkd2* FWD 5’- GGGGTCTCCTTCCATATCCAG-3’, *Prkd2* REV 5’-ACGATAGAACAGGCTAGTTGC-3’, *Prkd3* FWD 5’- GTCTGTCAAATGTATCTCTGCCA-3’, *Prkd3* REV 5’- GGTGAGTATGTGACTCTTCACTG-3’, *Il10* FWD 5’-CCCATTCCTCGTCACGATCTC-3’, *Il10* REV 5’-TCAGACTGGTTTGGGATAGGTTT-3’, *Tgfb1* FWD 5’-CTCCCGTGGCTTCTAGTGC-3’, *Tgfb1* REV 5’-GCCTTAGTTTGGACAGGATCTG-3’, *Pdcd1* FWD 5’-ACCCTGGTCATTCACTTGGG-3’, *Pdcd1* REV 5’-CATTTGCTCCCTCTGACACTG-3’, *Pdcd1l1* FWD 5’-GCTCCAAAGGACTTGTACGTG-3’, *Pdcd1l1* REV 5’-TGATCTGAAGGGCAGCATTTC-3’, *Il12b* FWD 5’-TGGTTTGCCATCGTTTTGCTG-3’, *Il12b* REV 5’-ACAGGTGAGGTTCACTGTTTCT-3’, *Il27a* FWD 5’-CTGTTGCTGCTACCCTTGCTT-3’, *Il27a* REV 5’-CACTCCTGGCAATCGAGATTC-3’.

### Statistical Analysis

Statistical analysis was performed using GraphPad Prism software and the statistical test used is indicated in the figure legends. P value of < 0.05 was considered statistically significant. The following denotations were used to further clarify statistical significance: ns=p>0.05, *=p<0.05, **=P<0.01, ***=p<0.005, ****=p<0.001. Unpaired student’s t-test was used to compare experiments with exactly two groups. One-way ANOVA was used to compare experiments with more than two groups. Two-way ANOVA was used to compare experiments with more than two groups tested under more than one condition. Sizes of animal or treatment groups are indicated in figure legends.

## Results

### BCR and TLR4 or CD40 Activation Is Sufficient to Drive Breg IL-35 Expression *Ex Vivo*

**Figure 1:**
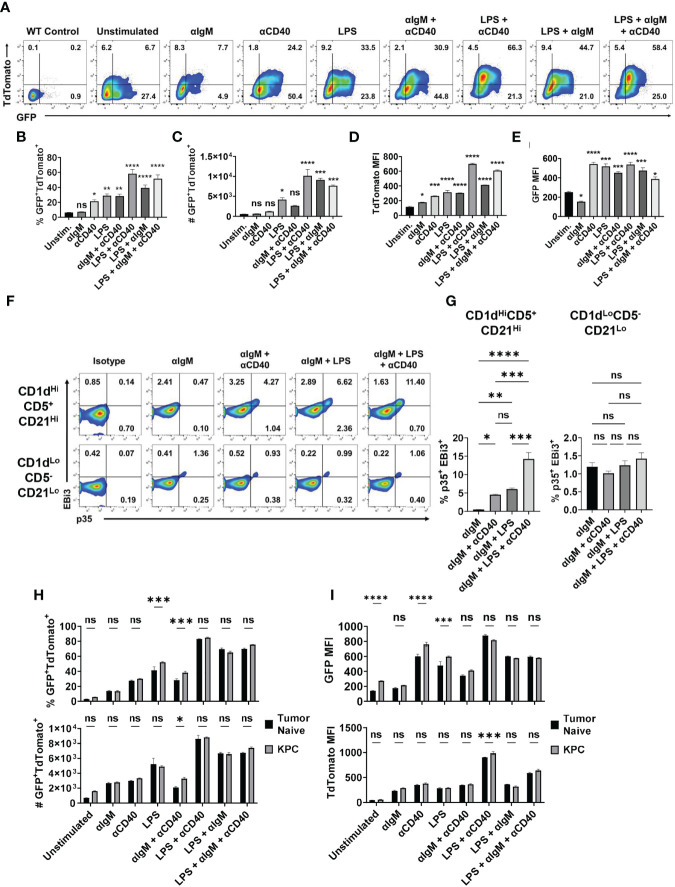
Combined BCR/TLR signaling induces IL-35 expression in B cells. **(A)** Representative flow cytometry plots of splenic *Il12a* ^GFP/GFP^; *Ebi3* ^Tom/Tom^ CD19^+^CD21^Hi^CD1d^Hi^CD5^+^ B cells after stimulation. **(B)** Frequency of GFP^+^TdTomato^+^ B cells from **(A)**. N=3. **(C)** Number of GFP^+^TdTomato^+^ B cells from **(A)**. N=3. **(D)** TdTomato mean fluorescent intensity (MFI) in B cells from **(A)**. N=3. **(E)** GFP mean fluorescent intensity (MFI) in B cells from **(A)**. N=3. **(F)** Representative flow cytometry plots of WT splenic CD19^+^CD21^Hi^CD1d^Hi^CD5^+^ (top) and CD19^+^CD21^Lo^CD1d^Lo^CD5^-^ (bottom) B cells intracellularly stained for p35 and EBi3 after stimulation. **(G)** Frequency of p35^+^EBi3^+^ B cells from **(F)**. N=3. **(H)** Frequency (top) and number (bottom) of GFP^+^TdTomato^+^ B cells from *Il12a* ^GFP/GFP^; *Ebi3* ^Tom/Tom^ B cells derived from tumor-naïve and KPC4662 tumor-bearing mice after *ex vivo* stimulation. N=3. **(I)** TdTomato mean fluorescent intensity (MFI) in B cells from **(H)** (top) and GFP MFI in B cells from **(H)** (bottom) after indicated *ex vivo* stimulation. N=3. Error bars indicate SEM. P values for 1B-E, G were calculated using one-way ANOVA. P values for 1H-I were calculated using two-way ANOVA. NS, non-significant, *p<0.05, **p<0.005, ***p<0.001, ****p<0.0001. Experiments were performed using 7–8-week-old mice of indicated genotypes. Experiments were repeated 3 times.

To understand how the expression of IL-35 subunits is regulated at single cell resolution, we generated a bona fide IL-35 reporter mouse by crossing our *Il12a*-GFP reporter mouse (18) to a previously generated and validated *Ebi3*-TdTomato reporter mouse (19). We tested the responsiveness of primary IL-35-reporter B cells to signals previously linked to either IL-10 and/or IL-35 expression *in vitro* (12, 17, 18). We previously identified the CD19^+^CD1d^Hi^CD21^Hi^CD5^+^ subset of B cells (regulatory B cells, Breg) as being the dominant B cell source of IL-35 expression in PDAC (5, 6). We utilized primary splenic CD19^+^CD1d^Hi^CD21^Hi^CD5^+^ B cells from IL-35 reporter mice for *ex vivo* stimulation assays featuring combinations of BCR, TLR4, and CD40 stimulation (**Figure 1A**). Using this model, we determined that BCR stimulation (αIgM) alone is not sufficient to induce IL-35 expression as the reporter signal intensity was not significantly increased after stimulation, due to fluctuation in the frequencies of individual TdTomato^+^ and GFP^+^ populations (**Figure 1B, C**). However, LPS stimulation or CD40 stimulation alone in the absence of a BCR signal was sufficient to expand a population of GFP^+^TdTomato^+^ Breg cells, with most significant changes in GFP MFI signal (**Figures 1B–E**). Combining LPS and CD40 stimulation led to the most significant increase in GFP^+^TdTomato^+^ cell frequency, number, and reporter signal intensity (**Figures 1B–E**). The use of αIgM instead of LPS as the main antigenic signal accompanying CD40 stimulation did generate a significant GFP^+^TdTomato^+^ cell population, but not to the same magnitude of LPS + αCD40 stimulation. Changes in the frequency of GFP^+^TdTomato^+^ reporter cells in the presence of LPS and CD40 activation were primarily driven by increase in TdTomato signal. We also examined the effect of combining BCR and TLR stimulation in the presence or absence of CD40 co-stimulation. Interestingly, combined BCR and TLR stimulation resulted in similar reporter signal intensity to LPS stimulation, but the combination resulted in significantly more double reporter-positive cells (p=0.02) and higher frequency (p=0.05) (**Figures 1B–E**). This suggests that BCR stimulation helps further drive the expansion of Bregs but requires TLR co-stimulation to do so.

Intracellular cytokine staining mirrored reporter findings to show that BCR stimulation alone was not sufficient to induce IL-35 (p35^+^EBi3^+^) expression in the regulatory B cell subset (**Supplementary Figures 1A, B** and **Figures 1F, G**). We observed a significantly lower percentage of stained p35^+^Ebi3^+^ cells compared to GFP^+^TdTomato^+^ B cells in each condition, but the trends in IL-35^+^ cell frequency was conserved between the two approaches. The addition of αCD40 stimulation to αIgM was able to induce a population of IL-35^+^ Breg cells (**Figures 1F, G**). Furthermore, BCR + LPS stimulation with or without CD40 co-stimulation was able to induce IL-35 expression in regulatory B cells, but not CD19^+^CD1d^Lo^CD21^Lo^CD5^-^ Bcon cells (**Figures 1F, G**). This suggests that *ex vivo*, BCR stimulation promotes the expansion of double positive regulatory cells only in the presence of either TLR and/or CD40 stimulation, and that inhibition of either the BCR or TLR signaling arm could potentially impact regulatory B cell function.

We also asked whether B cells derived from healthy mice would have the same responses to different stimuli than B cells from a tumor-bearing animal. When exposed to various stimuli *ex vivo*, splenic B cells derived from tumor-bearing mice generated a somewhat greater frequency of GFP^+^TdTomato^+^ B cells in response to LPS or αIgM + αCD40 compared to tumor-naïve B cells (**Figure 1H** and **Supplementary Figure 1C**). Examination of GFP and TdTomato signal intensity revealed that the increased frequency is due to GFP signal and not TdTomato (**Figure 1I**). The only instance we observed a significant increase in TdTomato signal intensity due to the influence of PDAC was with LPS + αCD40 stimulation, which did not have an increase in GFP intensity (**Figure 1I**). This is very interesting because *Ebi3* expression is more highly regulated of the two subunits, but PDAC is primarily modulating *Il12a* expression in the spleen. Overall, this does suggest that pancreatic tumors can pre-condition B cells to have augmented IL-35 responses to stimuli.

### BCR Engagement Promotes PDAC Growth and Represses Effector T Cell Responses

**Figure 2:**
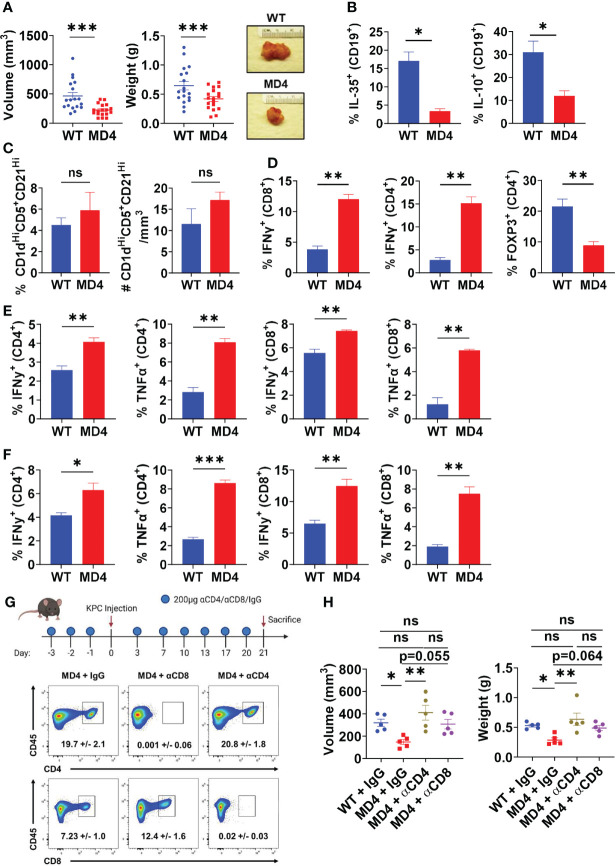
B cell receptor activation promotes pancreatic tumor growth, B cell IL-35 expression, and effector T cell activity. **(A)** Measured volumes and weights of orthotopic KPC4662 tumors from WT and MD4 mice 21 days post-tumor injection. N=18. **(B)** Frequencies of IL-35^+^ (p35^+^) and IL-10^+^ B cells isolated from WT and MD4 KPC4662 orthotopic tumors at day 21 post-tumor injection. N=3. **(C)** Frequency (left) and number (right) of CD19^+^CD21^Hi^CD1d^Hi^CD5^+^ B cells isolated from WT and MD4 KPC4662 orthotopic tumors at day 21 post-tumor injection. N=6. **(D)** Frequencies of and Foxp3^+^ and IFNγ^+^ CD4^+^ or CD8^+^ T cells isolated from KPC4662 orthotopic tumors of WT and MD4 tumor-bearing mice at day 21 post-tumor injection. N=3. **(E)** Frequencies of IFNγ ^+^ and TNFα^+^ CD4^+^ or CD8^+^ T cells isolated from spleens of WT and MD4 KPC4662 orthotopic tumor-bearing mice at day 21 post-injection. N=3. **(F)** Frequencies of IFN γ ^+^ and TNFα^+^ CD4^+^ or CD8^+^ T cells isolated from tumor-draining lymph nodes (TdLN) of WT and MD4 KPC4662 orthotopic tumor-bearing mice at day 21 post-injection. N=3. **(G)** Schematic of T cell depletion in MD4 mice with KPC4662 orthotopic tumors and representative flow cytometry plots of CD45^+^ splenocytes isolated at day 21 post-tumor injection. **(H)** Measured volumes and weights of orthotopic KPC4662 tumors from WT and MD4 mice with or without CD4^+^ or CD8^+^ T cell depletion 21 days post-tumor injection. N=5. Error bars indicate SEM; p values in **(A–F)** were calculated using unpaired t-test; p values in **(H)** were calculated using one-way ANOVA. NS, non-significant, *p<0.05, **p<0.005, ***p<0.001. Data are representative of three independent experiments. Experiments were performed using 7–8-week-old mice of indicated genotypes.

To understand the role of BCR activation in pancreatic tumor growth *in vivo*, we utilized the MD4 mouse model (30). B cells derived from MD4 mice have a fixed transgenic BCR that is reactive to hen egg lysozyme, therefore incapable of engaging host or pancreatic tumor-generated antigens. Autoimmune studies in MD4 mice linked disease remittance and inflammatory T cell activity to decreased suppressive abilities of B cells, thus we hypothesized that the inability to sense antigen may affect the suppressive abilities of B cells in PDAC (8). Tumor-naïve MD4 mice did not show any significant alterations in the development of immune cell populations or B cell subsets compared to WT mice (**Supplementary Figures 2A, B**). Syngeneic PDAC cells orthotopically injected into the pancreas of MD4 mice formed significantly smaller tumors than WT controls, indicating that BCR signaling can promote tumor growth (**Figure 2A**). Examination of the B cell infiltrate revealed a significant decrease in the frequency of IL-35^+^ and IL-10^+^ B cells in MD4 mice as compared to WT, indicating that BCR signaling in response to PDAC induces an immunosuppressive B cell phenotype *in vivo* (**Figure 2B** and **Supplementary Figure 3A**). The frequency of the Breg cell subset was not altered in MD4 tumors suggesting that the BCR signaling plays a role in regulatory B cell function and not expansion of the regulatory B cell subset in response to PDAC (**Figure 2C**). We recently showed that decrease in IL-35 production by B cells in PDAC results in significantly increased effector T cell responses coupled with decreased tumor growth (6). Consistent with this, we detected an increased frequency of IFNγ^+^CD4^+^ and IFNγ^+^CD8^+^ T cells in MD4 tumors coupled with a decreased frequency of Foxp3^+^ T cells compared to WT tumoral immune infiltrates (**Figure 2D** and **Supplementary Figure 3B**). Furthermore, we observed increased effector CD4^+^ and CD8^+^ T cell activity in both the spleen and draining lymph nodes of MD4 mice indicating the presence of a systemic response in secondary lymphoid organs as well (**Figures 2E, F** and **Supplementary Figures 3C, D**). To understand if decreased tumor growth in MD4 mice was dependent on T cell responses, we depleted CD4^+^ or CD8^+^ T cells (**Figure 2G**). Depletion of CD4^+^ T cells from MD4 mice resulted in significantly increased pancreatic tumor growth compared to control MD4 tumors, whereas depletion of CD8^+^ T cells showed a non-significant trend towards increase in tumor growth compared to isotype controls (**Figure 2H**). Collectively these data suggest that BCR activation promotes pancreatic cancer growth largely through suppression of CD4^+^ T cell responses *in vivo*.

### MyD88 Signaling in B Cells Does Not Impact IL-35^+^ Breg Frequency *In Vivo*

**Figure 3:**
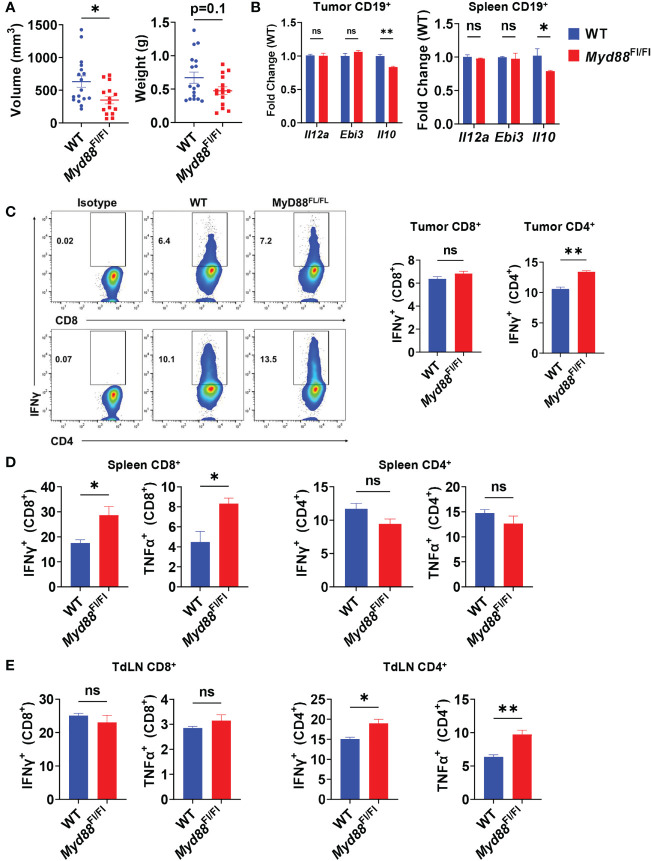
PDAC influences B cell IL-35 expression *in vivo* in a MyD88-independent manner. **(A)** Measured volumes and weights of orthotopic KPC4662 tumors from *Cd19* ^+/+^; *Myd88* ^Fl/Fl^ (WT) and *Cd19* ^Cre/+^; *Myd88* ^Fl/Fl^ (*Myd88* ^Fl/Fl^) mice at day 21 post-tumor injection. N=17. Cumulative of 3 individual experiments. **(B)** RT-PCR analysis of *Il12a*, *Ebi3*, and *Il10* expression from CD19^+^ B cells derived from tumors and spleens of *Cd19* ^+/+^; *Myd88* ^Fl/Fl^ and *Cd19* ^Cre/+^; *Myd88* ^Fl/Fl^ mice at day 21 post-tumor injection. N=5. **(C)** Intracellular flow cytometry of IFNγ and TNFα in CD8^+^ and CD4^+^ T cells derived from tumors of *Cd19* ^+/+^; *Myd88* ^Fl/Fl^ (WT) and *Cd19* ^Cre/+^; *Myd88* ^Fl/Fl^ (MyD88^Fl/Fl^) KPC4662 orthotopic tumor-bearing mice at day 21 post-tumor injection. N=4. **(D)** Intracellular flow cytometry of IFNγ and TNFα in CD4^+^ and CD8^+^ T cells derived from spleens of *Cd19* ^+/+^; *Myd88* ^Fl/Fl^ and *Cd19* ^Cre/+^; *Myd88* ^Fl/Fl^ KPC4662 orthotopic tumor-bearing mice at day 21 post-tumor injection. N=4. **(E)** Intracellular flow cytometry of IFNγ and TNFα in CD4^+^ and CD8^+^ T cells derived from spleens of *Cd19* ^+/+^; *Myd88* ^Fl/Fl^ and *Cd19* ^Cre/+^; *Myd88* ^Fl/Fl^ KPC4662 orthotopic tumor-bearing mice at day 21 post-tumor injection. N=4. Error bars indicate SEM; p values in **(A–D)** were calculated using t-test; p values in **(B)** were calculated using two-way ANOVA. NS, non-significant, *p<0.05, **p<0.005, ***p<0.001 Experiments were performed using 7–8-week-old mice of indicated genotypes.

TLR-mediated B cell suppression of T cell responses in models of bacterial infection and autoimmune disease has been linked mainly to expression of IL-10 (10, 31), and is highly dependent on signaling through the TLR adapter protein MyD88. Recently, TLRs have also been discovered to induce IL-35 expression in B cells in the same disease models (12), leading us to question the roles of TLRs on B cells in PDAC progression and T cell suppression. To understand the role of B cell specific TLR activation in pancreatic tumor growth *in vivo*, we generated *CD19^Cre/+^; Myd88^Fl/Fl^* mice. Healthy mice with *Myd88* recombination specifically in B cells did not have altered immune cell development or B cell development, congruent with a similar *Cd79a^Cre^; Myd88^Fl/Fl^* model (**Supplementary Figures 1A, B**) (32). Orthotopic pancreatic tumors in *CD19^Cre/+^; Myd88^Fl/Fl^* mice displayed trends towards decreased growth as compared to *CD19^+/+^; Myd88^Fl/Fl^* controls, but the change in growth was less robust than the tumor growth change displayed in MD4 mice (**Figures 3A**, **2B**). We observed that IL-10 expression in B cells was significantly downregulated in *CD19^Cre/+^; Myd88^Fl/Fl^* mice (**Figure 3B**). However, expression of neither IL-35 subunits was affected, indicating that B cell expression of IL-10 is at least partially dependent on MyD88 in PDAC, but IL-35 could primarily be regulated by another antigenic pathway, most likely the BCR (**Figure 3B**). The loss of MyD88 signaling in B cells resulted in increased CD8^+^ T cell effector responses in the spleen but not in the tumor nor the tumor-draining lymph node upon tumor challenge, suggesting insufficient T cell priming (**Figures 3C–E**). Inversely, CD4^+^ T cell effector responses were significantly increased in the tumor and tumor-draining lymph node of *CD19^Cre/+^; Myd88^Fl/Fl^* mice and unaltered in the spleen (**Figures 3C–E**). Collectively these data demonstrate that MyD88 signaling in B cells does not play a prominent role in promoting PDAC growth.

### BCR Pathway Kinase Inhibition Represses IL-35 Expression

**Figure 4:**
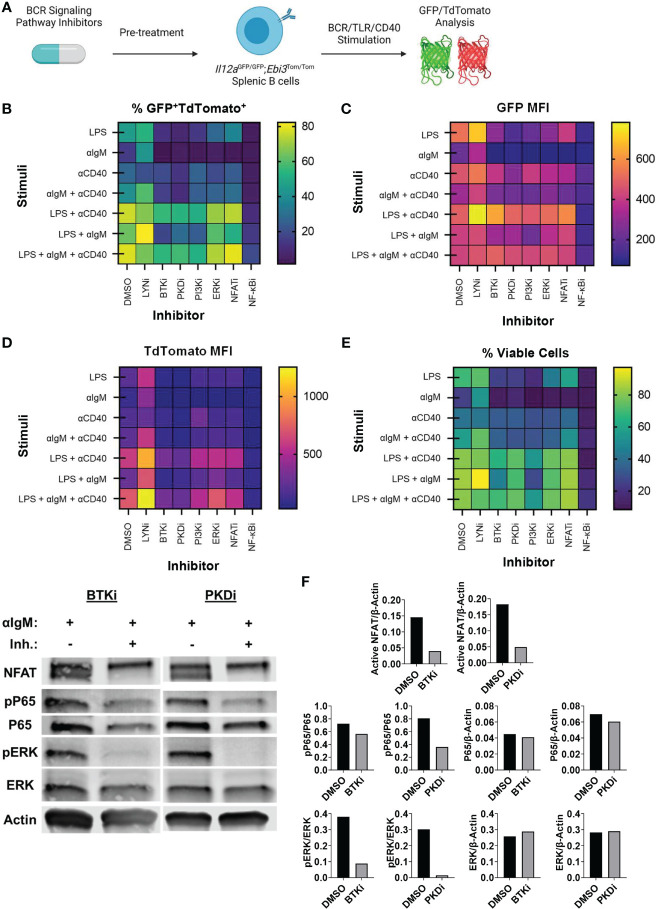
Identification of BCR pathway signaling molecules that govern IL-35 expression. **(A)** Heatmap displaying frequency of GFP^+^TdTomato^+^ B cells from all stimulation conditions and inhibitors 48 hours after stimulation/inhibition. N=3 per condition. Experiment repeated 2 times. **(B)** Heatmap displaying frequency of viable B cells from all stimulation conditions and inhibitors 48 hours after stimulation/inhibition. N=3 per condition. Experiment repeated 2 times. **(C)** Heatmap displaying TdTomato mean fluorescent intensity (MFI) from all stimulation conditions and inhibitors 48 hours after stimulation/inhibition. N=3 per condition. Experiment repeated 2 times. **(D)** Heatmap displaying GFP mean fluorescent intensity (MFI) from all stimulation conditions and inhibitors 48 hours after stimulation/inhibition. N=3 per condition. Experiment repeated 2 times. **(E)** Western immunoblots of WT splenic B cells treated with DMSO, BTKi (5uM), or PKDi (10uM) for 30 minutes followed by stimulation with aIgM (10µg/mL). 30ug of total protein loaded per lane. **(F)** Quantification of immunoblots in **(E)**.

Since BCR signaling was dominant in controlling pancreatic tumor growth *in vivo*, we examined how key signaling molecules in the BCR signaling pathway could contribute to IL-35 expression. To understand how the BCR could affect IL-35 expression we performed a screen with small molecule inhibitors of the BCR signaling pathway using primary IL-35-reporter B cells isolated from *Il12a* ^GFP/GFP^; *Ebi3* ^Tom/Tom^ spleens (**Figure 4A**). Inhibitor efficacy was assessed by Western blotting (**Supplementary Figure 4A**) and analyzed for reporter fluorescence *via* flow cytometry. We reasoned that the effector pathways that may most significantly affect IL-35 production may include pathways that promote cross talk between BCR and CD40 signaling, mimicking B cell and T cell interactions *in vivo*. Protein Kinase D (PKD) is a serine/threonine kinase that in response to BCR stimulation promotes synergy between the BCR and CD40 in a TRAF2-dependent manner (33, 34). PKD is required in B cells for complete proliferation, IL-6, TNFα, and IgM secretion responses from antigen and CD40 co-stimulation (33, 34). We tested the role of PKD in IL-35 expression as well as upstream BCR kinases Bruton’s tyrosine kinase (Btk), Phosphoinositide 3-kinase delta (PI3Kδ), and Lyn. Additionally, we tested key downstream signaling effectors: extracellular signal-regulated kinase 1/2 (ERK1/2), Nuclear factor of activated T-cells (NFAT), and nuclear factor kappa-light-chain-enhancer of activated B cells (NF-κB) in immunosuppressive cytokine production. We chose to investigate the roles of these effectors in the context of not only BCR stimulation, but also TLR (LPS) stimulation as well, since recent work demonstrated that effective TLR signaling activates and requires components of the BCR signaling pathway to fully signal (35).

We observed significant decreases in the frequency of GFP^+^TdTomato^+^ B cells when several kinases were individually inhibited (**Figure 4B**). Additionally, we observed significant changes in the MFI of GFP and TdTomato reporter signal with different inhibitors (**Figures 4C, D**). Inhibition of the BCR signaling phosphatase Lyn did not result in significant alteration of GFP or TdTomato intensity in response to all stimuli and significantly increased activity in most instances where BCR (αIgM) stimulation was used (**Figures 4B–D** and **Supplementary Figures 6D, 7D**). This is likely due to Lyn’s role in the negative regulation of the BCR signaling pathway and is consistent with previous data showing expansion of immunosuppressive IL-10 producing B cells in Lyn^-/-^ mice (36). Inhibition of BCR signaling kinases PKD, PI3Kδ and Btk, on the other hand, resulted in the most significant downregulation of GFP^+^TdTomato^+^ cell frequency and TdTomato signal intensity in almost all stimulation conditions (**Figures 4B–D** and **Supplementary Figures 5A-C, 6A-C**). In conditions containing αIgM stimulation with these kinase inhibitors, PKDi was the most efficient at reducing GFP^+^TdTomato^+^ cell frequency and TdTomato signal intensity (**Figures 4B–D** and **Supplementary Figures 5A-C, 6A-C**). At the same time, Btk or PI3Kδ inhibition was more toxic than PKD inhibition in αIgM stimulation conditions (**Figure 4E** and **Supplementary Figures 4B, C**). This is likely related to the wider range of signaling pathways supporting survival that are activated downstream of Btk or PI3Kδ than those downstream of PKD. Overall, these observations suggest that PKD inhibition may be the most promising therapeutic target for IL-35 inhibition.

With regards to the signaling effector pathways downstream of BCR and CD40 signaling, the most prominent effect on reporter signal intensity was seen with inhibition of NF-κB signaling. Inhibition of IKKα/β (NF-κBi) resulted in near complete abolition of both GFP and Tomato signals regardless of stimulation conditions (**Supplementary Figures 5E, 6E, 7E**). This agrees with prior reports stating the requirement of NF-kB signaling in the expression of *Ebi3* in B cells (37) and *Il12a* in antigen presenting cells (38). However, NF-kB inhibition *in vitro* over 48 hours resulted in high cellular toxicity regardless of the stimulation condition, thus making it an unlikely candidate for therapeutic use. Inhibition of ERK1/2 did not result in significant alterations in GFP or TdTomato reporter signal intensity (**Supplementary Figures 6F, 7F**). ERK1/2 inhibition did however decrease the frequency of GFP^+^TdTomato^+^ B cells leading us to conclude that ERK signaling does not play a role in regulating IL-35 expression, but rather aids in the proliferation of IL-35 expressing cells (**Supplementary Figure 5F**). This is consistent with the effect of MEK inhibition on IL-10^+^ regulatory B cell expansion (39). Inhibition of NFAT signaling, which regulates BCR-dependent IL-10 expression (40), resulted in significant decreases in GFP^+^TdTomato^+^ cell frequency primarily through inhibition of TdTomato reporter intensity (**Supplementary Figure 5G, 6G**). The regulation of *Ebi3* transcription by NFAT is entirely plausible as there is an NFAT binding region in the *Ebi3* promoter region but was determined to be non-essential for activation in BMDCs (37). We continued to evaluate the role of PKD in B cell signaling by examining the effects on ERK, NFAT, and NF-kB activation in comparison to Btk inhibition, which achieves similar IL-35 reporter inhibition (**Figure 4F**). Inhibition of Btk with Ibrutinib and PKD with CRT0066101 (41) resulted in significant inhibition of ERK, NFAT, and NF-kB signaling to relatively similar extents (**Figure 4G**). Collectively, this data points to IL-35 expression, with inhibition of PKD pathway being the most efficient in shutting down expression of IL35 and maintaining cellular viability.

### Protein Kinase D2 Function in B Cells Promotes IL-35 Expression and PDAC Growth

**Figure 5:**
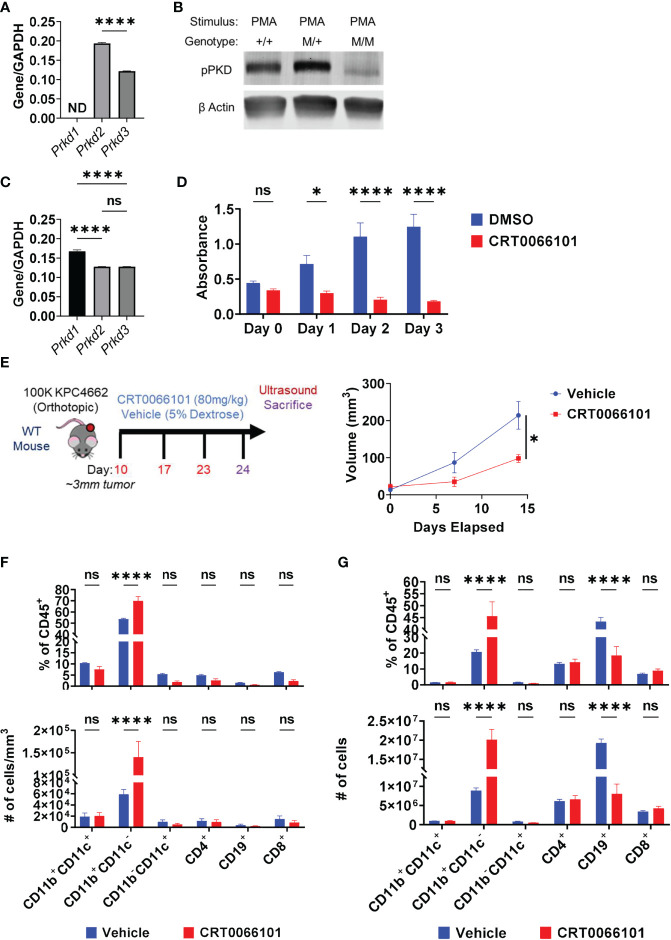
Protein Kinase D regulates PDAC growth in immunocompetent mice. **(A)** RT-PCR analysis of PKD isoform expression in WT splenic B cells. ND=not detectable. N=3. **(B)** Phospho-PKD (S744/748) immunoblot of PMA-stimulated splenic B cells that are WT (+/+), heterozygous (M/+), or homozygous (M/M) for S707A/S711A mutation in *Prkd2*. **(C)** RT-PCR analysis of PKD isoform expression in KPC4662 cells. N=3. **(D)** MTT proliferation assay of DMSO or CRT0066101 treated KPC4662 cells *in vitro*. N=5. **(E)** Treatment schema and study design (left). Briefly, 10 days post-KPC4662 injection, WT mice were stratified into groups and treated with CRT0066101 or vehicle QOD by oral gavage. Mice were treated for 2 weeks before sacrifice at day 24 post-tumor injection. Orthotopic KPC4662 tumor volumes of CRT0066101 and vehicle treated mice as measured by ultrasound imaging (right). **(F)** Frequencies of indicated CD45^+^ immune populations isolated from tumors (top) and cell number per mm^3^ of tumor (bottom) from vehicle and CRT0066101 treated mice. **(G)** Frequencies of indicated CD45^+^ immune populations isolated from tumors (top) and cell number (bottom) from vehicle and CRT0066101 treated mice. Error bars indicate SEM; p values in **(A, E)** were calculated using unpaired t-test; p values in **(C)** were calculated using one-way ANOVA; p values in **(D, F, G)** were calculated using two-way ANOVA. NS, non-significant, *p<0.05, **p<0.005, ***p<0.001 ****p<0.0001. Experiments were performed using 7–8-week-old mice of indicated genotypes.

We focused our efforts on further evaluating the role of PKD in IL-35 expression by B cells. CRT0066101 (PKDi) inhibits all 3 PKD isoforms, but the relative contributions of each isoform on IL-35 expression is unknown. In B lymphocytes, the PKD2 isoform is predominantly expressed (42) whereas PKD3 is expressed at lower levels and PKD1 expression was not detected by RT-PCR analysis (**Figure 5A**). Next, we aimed to understand the relative contributions of PKD2 and PKD3 in B cell signaling. To do this, we used B cells isolated from a mouse model harboring a knock-in of S707A and S711A mutations into the WT *Prkd2* locus, which result in a lack of PKD2 catalytic activity (42). Therefore, B cells derived from this model only have active PKD3 signaling. PMA stimulation of homozygous *Prkd2-*S707A/S711A splenic B cells resulted in an almost complete absence of pan-PKD serine phosphorylation in the catalytic domain leading us to conclude that PKD2 and not PKD3 is the dominant active PKD isoform in primary B cells (**Figure 5B**). PKD signaling is not only active in B cells but is also active in Kras mutant pancreatic cancer cells (43). Prior studies revealed PKD function significantly impacts pancreatic cancer cell growth and metastatic potential (41, 44). RT-PCR analysis revealed that *KPC* cells express all PKD isoforms, predominantly PKD1, which is consistent with other PDAC cell lines (41, 45, 46) (**Figure 5C**). To understand the role of PKD signaling in KPC4662, we used CRT0066101 and performed MTT proliferation assays *in vitro* (**Figure 5D**). Consistent with other PDAC lines previously tested (41), KPC cell proliferation is significantly inhibited with loss of PKD function (**Figure 5D**).

*In vivo*, we observed a significant delay in orthotopic tumor growth of immunocompetent mice treated with CRT0066101 inhibitor (**Figure 5E**). However, this experiment could not discern the potential contribution of PKD inhibition in cancer cells versus immune cells on tumor growth. To parse apart the effects of immune PKD function from cancer-cell autonomous PKD function in PDAC, we orthotopically injected KPC4662 cells into *Prkd2*-S707A/S711A mice. We observed no significant changes in tumor growth in the absence of host PKD2 function (**Supplementary Figure 8A**) suggesting that cancer cell PKD function is the key driver of PDAC tumor growth. However, we hypothesized that inhibition of PKD function in T cells, may have hindered anti-tumor immunity, resulting in increased tumor growth. PKD contributes to the expression of the effector cytokine IFNγ in response to TCR stimulation (42, 47). We also observed that activated *Prkd2*-S707A/S711A CD4^+^ and CD8^+^ T cells have significantly reduced IFNγ production *ex vivo* suggesting that T cell PKD function also plays a significant role in tumor growth (**Supplementary Figures 9A, B**). Regarding B cells, examination of the tumor immune infiltrate in both PKDi and *Prkd2*-S707A/S711A tumors by flow cytometry revealed that the frequency of infiltrating B cells was significantly decreased (**Figure 5F**) (**Supplementary Figure 8B**). Inversely, we observed a substantial increase in the frequency of CD11b^+^ myeloid cells upon PKD pharmalogical or genetic inhibition (**Figure 5F**) (**Supplementary Figure 8B**). Suppression of PKD in myeloid cells prevents pro-inflammatory cytokine expression in response to TLR9 agonists (48) and bacterial infection (49), but the effects in response to cancer are not known. CD4^+^ and CD8^+^ T cell frequencies were not decreased with PKDi, but CD19^+^ B cell frequencies and overall cell number were significantly reduced (**Figure 5G**). We further examined if the decreased tumoral B cell infiltrate was attributable to a specific B cell subset and found that PKD inhibition primarily decreased the frequency of follicular (CD19^+^CD93^-^CD21^Lo^IgD^Hi^) B cells within the tumor (**Supplementary Figure 10A**). Collectively, this data suggests that the decrease in tumor growth is due to inhibition of cancer-cell autonomous PKD, and the role of B cell and/or T cell PKD signaling in PDAC tumor immunity needs to be further understood *in vivo*.

**Figure 6:**
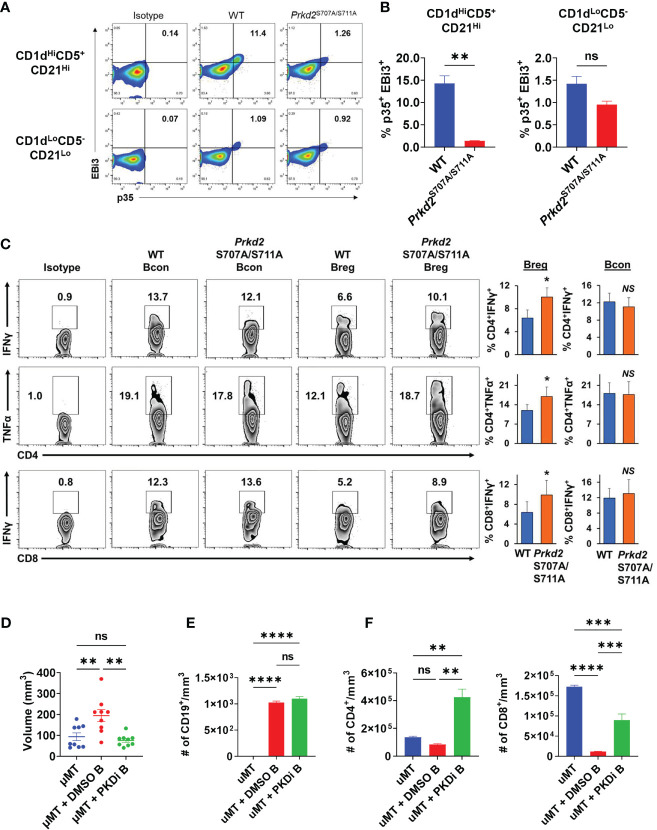
Inhibition of B cell PKD function suppresses IL-35 expression and tumor growth. **(A)** Representative flow cytometry plots of intracellular EBi3 and p35 in WT and homozygous *Prkd2* ^S707A/S711A^ CD19^+^CD21^Hi^CD1d^Hi^CD5^+^ and CD19^+^CD21^Lo^CD1d^Lo^CD5^-^ splenic B cells stimulated for 48 hours with LPS + αIgM + αCD40 *ex vivo*. N=4. **(B)** Frequency of p35^+^EBi3^+^ B cells from **(A)**. N=4. **(C)** Intracellular flow cytometry of IFNγ and TNFα from CD4^+^ and CD8^+^ T cells co-cultured in contact-independent transwells with either WT or homozygous *Prkd2* ^S707A/S711A^ CD19^+^CD21^Hi^CD1d^Hi^CD5^+^ regulatory B cells. N=3. **(D)** Orthotopic KPC4662 tumor volumes of µMT mice reconstituted with DMSO or CRT0066101 (PKDi) treated WT splenic B cells. N=9. Cumulative of 2 independent experiments. **(E)** Quantification of intratumoral CD19^+^ B cells per mm^3^ of tumor by flow cytometry from mice in **(D)**. N=5. **(F)** Quantification of intratumoral CD4^+^ T cells (left) and CD8^+^ T cells (right) per mm^3^ of tumor by flow cytometry from mice in **(D)**. N=5. Error bars indicate SEM; p values in **(B, C)** were calculated using unpaired t-test; p values in **(D–F)** were calculated using one-way ANOVA; p values in **(G)** were calculated using two-way ANOVA. NS, non-significant, *p<0.05, **p<0.005, ***p<0.001, ****p<0.0001. Experiments were performed using 7–8-week-old mice of indicated genotypes.

To elucidate the effects of PKD2 function on IL-35 specifically in the regulatory B cell subset, we stimulated WT and *Prkd2^S707A/S711A^* CD19^+^CD1d^Hi^CD21^Hi^CD5^+^ Bregs *in vitro* and analyzed p35 and EBi3 expression by flow cytometry (**Figure 6A**). Deficiency in PKD2 function led to a significant decrease in the frequency of p35^+^EBi3^+^ (IL-35^+^) Breg cells, confirming that PKD2 is a critical regulator of IL-35 expression in regulatory B cells (**Figure 6B**). Furthermore, we tested how deficiency in PKD2 function affected the expression of other IL-12 family cytokines, IL-12 and IL-27. *Il27a* and *Il12b* are not widely expressed by regulatory B cells (5). PKD2 functional deficiency does not enhance expression of Il12b under varying stimulation conditions, but it does significantly enhance *Il27a* under BCR and CD40 stimulation suggesting that PKD2 represses IL-27 production by regulatory B cells in certain conditions (**Supplementary Figure 11A**). We also tested the expression of other immunosuppressive cytokines, *Il10* and *Tgfb1* in the context of PKD2 functional deficiency. While *Tgfb1* expression is not altered with PKD2 function, *Il10* expression was significantly enhanced in certain stimulation conditions indicating a potential role for PKD2 in regulating IL-10 as well (**Supplementary Figure 11B**). To test the functional ability of *Prkd2^S707A/S711A^* Bregs to suppress T cell function we established *in vitro* transwell co-culture assays, containing activated WT and *Prkd2*-S707A/S711A CD19^+^CD1d^Hi^CD21^Hi^CD5^+^ Bregs and activated WT CD4^+^ and CD8^+^ T cells (**Figure 6C**). *Prkd2^S707A/S711A^* regulatory B cells were significantly less effective at suppressing effector T cell cytokine expression in both CD4^+^ and CD8^+^ T cells (**Figure 6C**).

To understand the role of B cell specific PKD2 function on tumor growth we reconstituted mice lacking mature B cells (µMT) with splenic B cells treated *ex vivo* with either CRT0066101 (PKDi) or DMSO followed by orthotopic injection of *KPC* cells. Mice reconstituted with PKDi-treated B cells had significantly smaller tumors than mice reconstituted with DMSO-treated B cells (**Figure 6D**). Examination of the tumor infiltrating lymphocytes revealed that the number of B cells in tumors was not altered by PKD inhibition (**Figure 6E**). However, PKD inhibition in B cells led to a significant increase in intratumoral CD4^+^ and CD8^+^ T cells as compared to DMSO controls (**Figure 6F**). Collectively this data suggests that PKD2 plays an essential role in not only IL-35 expression by B cells, but also effector T cell function and ultimately regulates balance of tolerogenic and anti-tumor immunity in pancreatic tumor growth.

## Discussion

We have identified BCR signaling as an important driver in establishment of IL35^+^ regulatory B cell function in pancreatic cancer. Our data suggests that while both BCR and MyD88 signaling in B cells promote IL-35 expression *in vitro*, BCR signaling plays a dominant role in IL-35 expression *in vivo* in response to pancreatic cancer. We previously observed the immunological effects of targeting IL-35 in PDAC and concluded that IL-35 inhibition potently increases effector T cell responses and reduces tumor growth (5, 6). In comparison, inhibited BCR signaling *in vivo* (**Figure 2A**) results in similar potent increases in effector T cell responses and decreased tumor growth (**Figures 2C–F**). Inhibition of MyD88 signaling however, did not lead to potent T cell suppression both *in vivo* and *in vitro* (**Figures 3C–F**). This enforces the fact that B cell suppression of T cells in PDAC is primarily BCR-mediated.

One possibility is that MyD88 is not the key regulator of IL-35 expression in response to TLR stimuli. While all TLRs except TLR3 signal through MyD88, TLR4 also signals through TRIF to mediate downstream gene expression that is dependent on IRF3. *Trif* ^-/-^ murine dendritic cells express significantly less *Ebi3* after LPS stimulation than WT controls (50), so B cells may also require TRIF for *Ebi3* expression. TLR stimulation also requires components of the BCR signaling pathway to fully transduce gene expression as well. For example, *Il10* and *Il6* expression was significantly decreased in *Syk*-deficient B cells after stimulation with LPS demonstrating that TLR4 signaling is not dependent on MyD88 alone (35). Activation of the BCR pathway kinases through TLR stimulation may explain the role of TLR signaling in response to pancreatic cancer. Additionally, the BCRs ability to capture antigens could be acting as a priming mechanism for TLR signaling. Intracellular TLR responses are highly dependent on BCR activity if nothing more than to shuttle TLR antigens such as DAMPs to TLR-containing endosomes. BCR recognition of apoptotic cells is required to induce-TLR9 mediated IL-10 expression in B cells and enhances TLR4-mediated IL-10 expression (15). We primarily focused on TLR4, but the role of intracellular TLR stimulation in B cell-mediated immunosuppression will be important to investigate in the future.

Recently a few studies have looked at the role of BCR signaling proteins to understand their relevance in regulatory B cell biology. Inhibition of the kinase MEK in BCR stimulated B cells inhibited the development of regulatory B cells, but the effect on suppressive cytokine production is not known (39). Similarly, inhibition of Bruton’s tyrosine kinase (Btk), a central kinase in the BCR signaling pathway, inhibits regulatory B cell development and IL-35 mRNA expression *in vitro* (51). However, this finding was performed in the context of αCD40, IL-6, and IL-1β activating Btk so the role of the BCR in this process is still unknown. A key point of emphasis in future studies will be to understand the composition of the BCR in regulatory B cells. It is not yet known whether these BCRs are directed in a tumor-antigen specific manner or are more host-reactive in nature like the BCRs of marginal zone and B-1 B cells. A key regulator of IL-35 expression *in vivo* we did not examine is CD40. TLR stimulation alone produces significantly less IL-35 *in vitro* than TLR and CD40 co-stimulation (12, 18). Furthermore, B cell expression of CD40 is also required for protection in models of collagen-induced arthritis, ulcerative colitis, EAE, and salmonella infection (7–9, 12, 52). CD40 stimulation to regulatory B cells is derived from cognate CD4^+^ T cells in EAE (52), but it is not known in pancreatic cancer where antigen-specific T cell responses are not as prevalent. Deletion of CD40 in B cells harboring PDAC tumors, could theoretically result in two opposing results. Loss of CD40 in regulatory B cells would hypothetically inhibit their expansion, but loss of CD40 in effector B cell populations would inhibit their maturation as well.

We demonstrate that Protein Kinase D2 function in B cells is essential for expression of IL-35 and promotes pancreatic tumor growth. Pancreatic cancer cell-specific PKD function is important for cell proliferation, so the double-edged effect of inhibiting B cell and PDAC cell PKD makes it a promising target heading forward. Previous PDAC model studies *in vivo* utilized immunocompromised mice for evaluation of PKD inhibition (41). We have expanded that knowledge by inhibiting PKD function in immunocompetent PDAC models and discovered that tumor growth remains delayed. However, PKD inhibition seems to affect more than B cells in the immune system as T cell effector function is highly dependent on PKD function. Targeting PKD inhibitors to specific cell types will be key for future therapeutic approaches.

## Data Availability Statement

The raw data supporting the conclusions of this article will be made available by the authors, without undue reservation.

## Ethics Statement

The animal study was reviewed and approved by Division of Comparative Medicine, The University of North Carolina at Chapel Hill.

## Author Contributions

DM and YP-G contributed to the conception and design of the study. DM, BM, and YP-G developed the methodology of the study. DM, BM, and CS contributed to the acquisition of all data, data quality assessment, and data analysis in the study. DM and YP-G contributed to the interpretation of the data. DM and YP-G were responsible for writing the manuscript. All authors contributed to the article and approved the submitted version.

## Funding

This work was supported in part by R37 CA230786 (YP-G), University Cancer Research Fund at the University of North Carolina at Chapel Hill, United States (YP-G); Concern Foundation Conquer Cancer Now Award (YP-G), F31 CA239494 (DM), and T32 CA71341-20 (DM). The UNC Flow Cytometry Core Facility and the UNC Lineberger Animal Studies Core are supported in part by P30 CA016086 Cancer Center Core Support Grant to the UNC LCCC.

## Conflict of Interest

The authors declare that the research was conducted in the absence of any commercial or financial relationships that could be construed as a potential conflict of interest.

## Publisher’s Note

All claims expressed in this article are solely those of the authors and do not necessarily represent those of their affiliated organizations, or those of the publisher, the editors and the reviewers. Any product that may be evaluated in this article, or claim that may be made by its manufacturer, is not guaranteed or endorsed by the publisher.
