# Supplementary material for: B Cell Receptor Signaling and Protein Kinase D2 Support Regulatory B Cell Function in Pancreatic Cancer
Source: Front Immunol. 2022 Jan 3;12:745873. doi: 10.3389/fimmu.2021.745873 (PMC8761795; doi:10.3389/fimmu.2021.745873)
Supplement: Supplementary file 1 [file Presentation_1.pptx]

## Slide 1
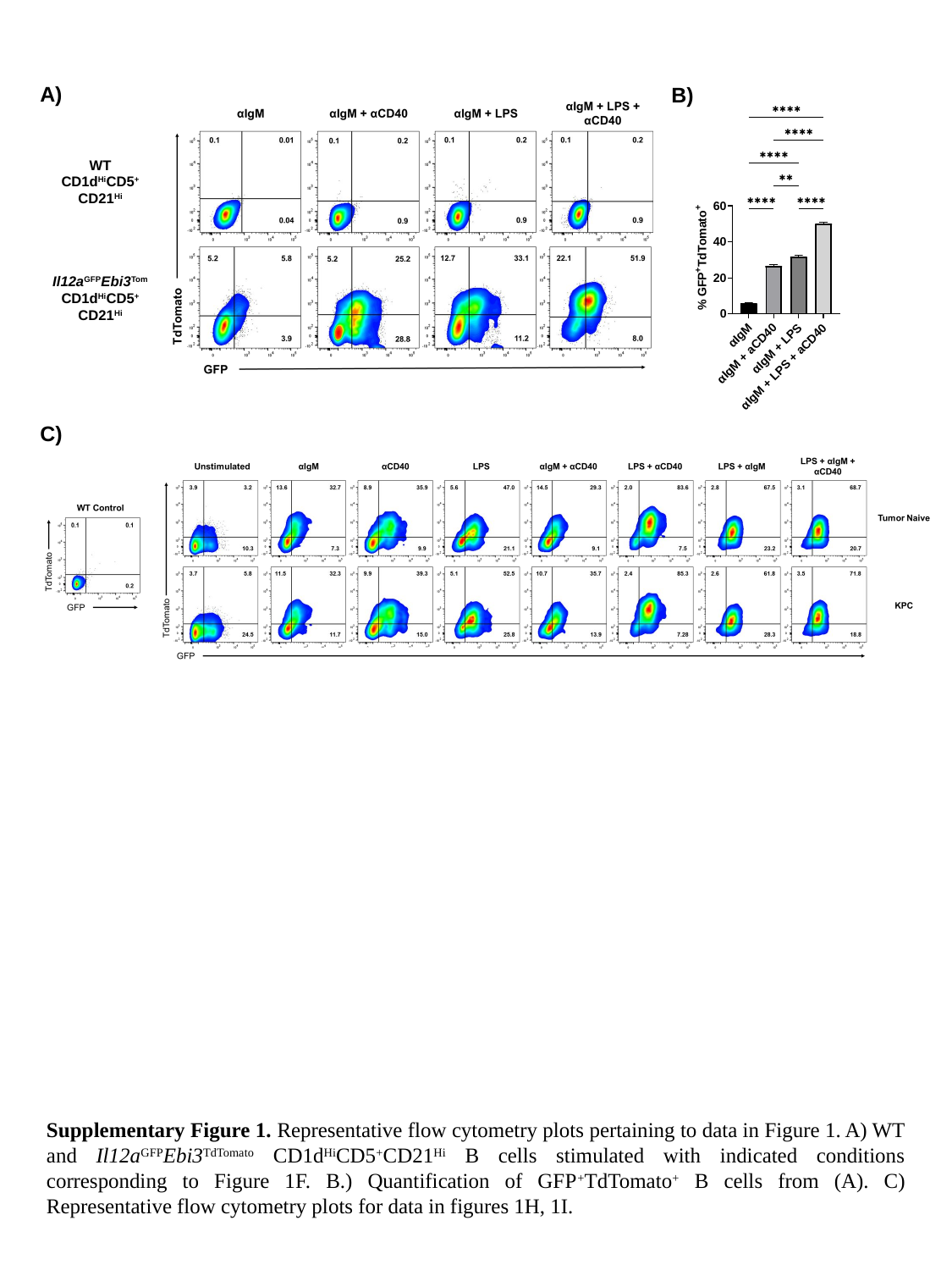

A)
B)
WT
CD1dHiCD5+
CD21Hi
Il12aGFPEbi3Tom
CD1dHiCD5+
CD21Hi
C)
Supplementary Figure 1. Representative flow cytometry plots pertaining to data in Figure 1. A) WT and Il12aGFPEbi3TdTomato CD1dHiCD5+CD21Hi B cells stimulated with indicated conditions corresponding to Figure 1F. B.) Quantification of GFP+TdTomato+ B cells from (A). C) Representative flow cytometry plots for data in figures 1H, 1I.

## Slide 2
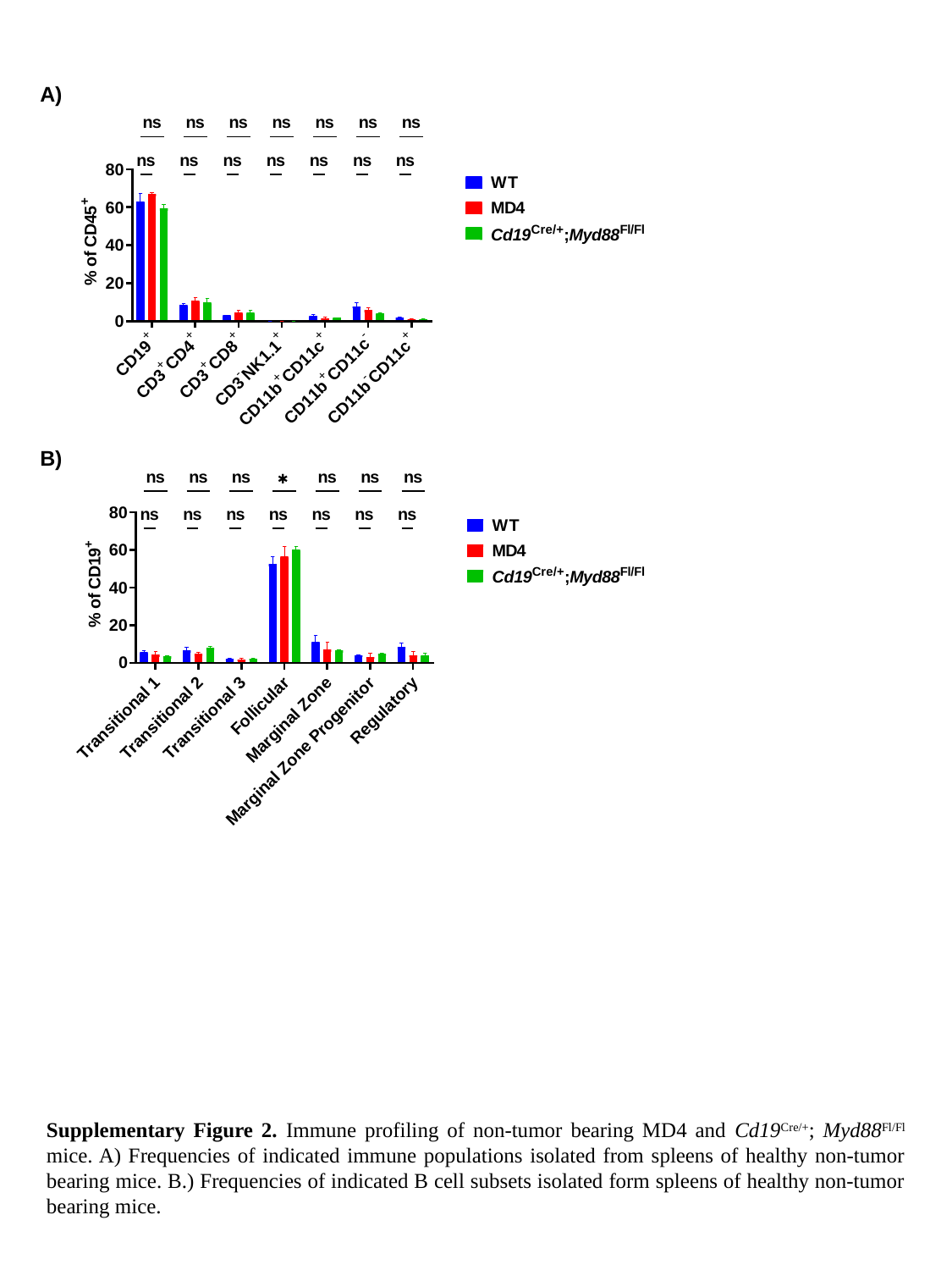

A)
B)
Supplementary Figure 2. Immune profiling of non-tumor bearing MD4 and Cd19Cre/+; Myd88Fl/Fl mice. A) Frequencies of indicated immune populations isolated from spleens of healthy non-tumor bearing mice. B.) Frequencies of indicated B cell subsets isolated form spleens of healthy non-tumor bearing mice.

## Slide 3
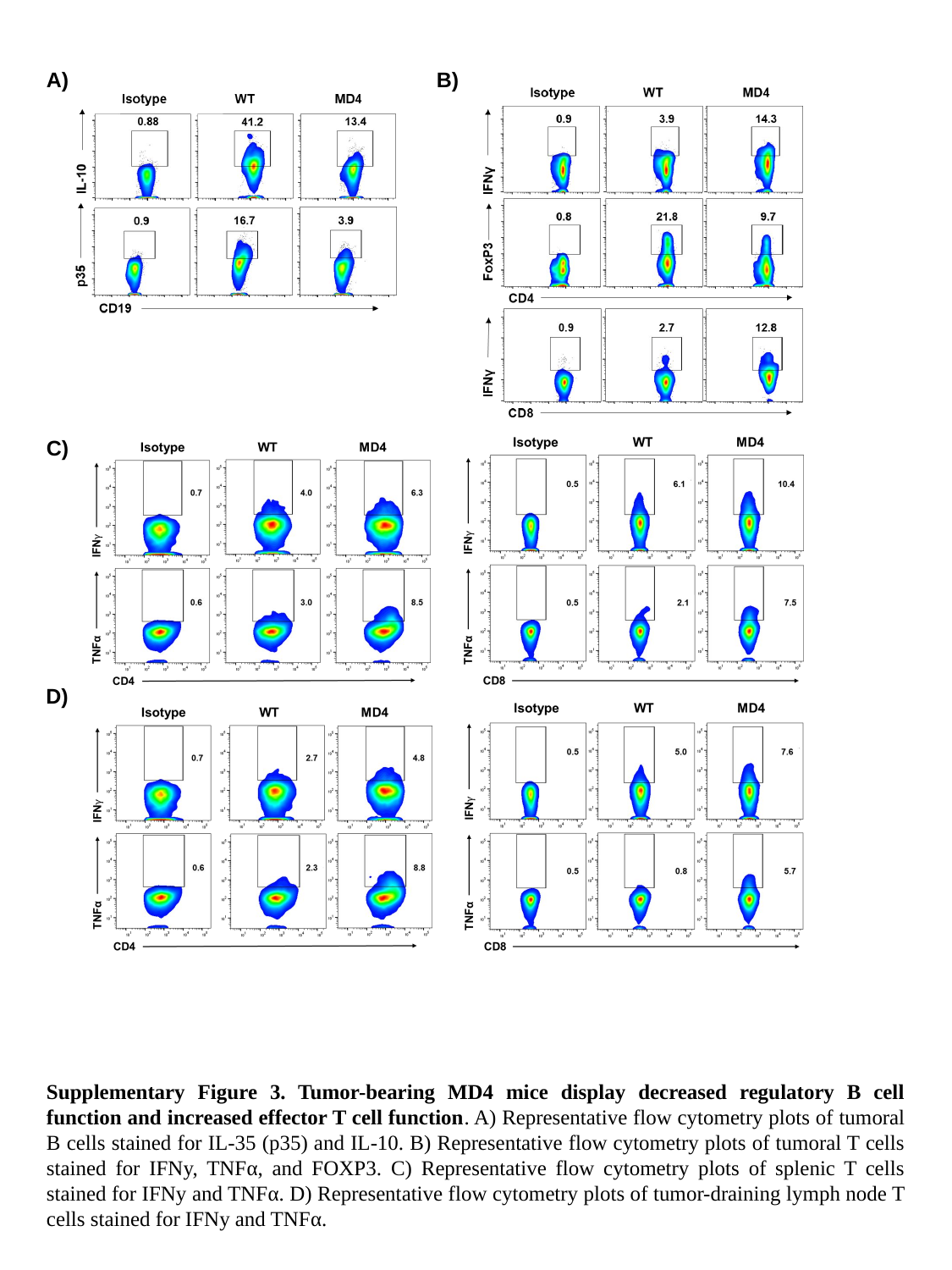

A)
B)
C)
D)
Supplementary Figure 3. Tumor-bearing MD4 mice display decreased regulatory B cell function and increased effector T cell function. A) Representative flow cytometry plots of tumoral B cells stained for IL-35 (p35) and IL-10. B) Representative flow cytometry plots of tumoral T cells stained for IFNy, TNFα, and FOXP3. C) Representative flow cytometry plots of splenic T cells stained for IFNy and TNFα. D) Representative flow cytometry plots of tumor-draining lymph node T cells stained for IFNy and TNFα.

## Slide 4
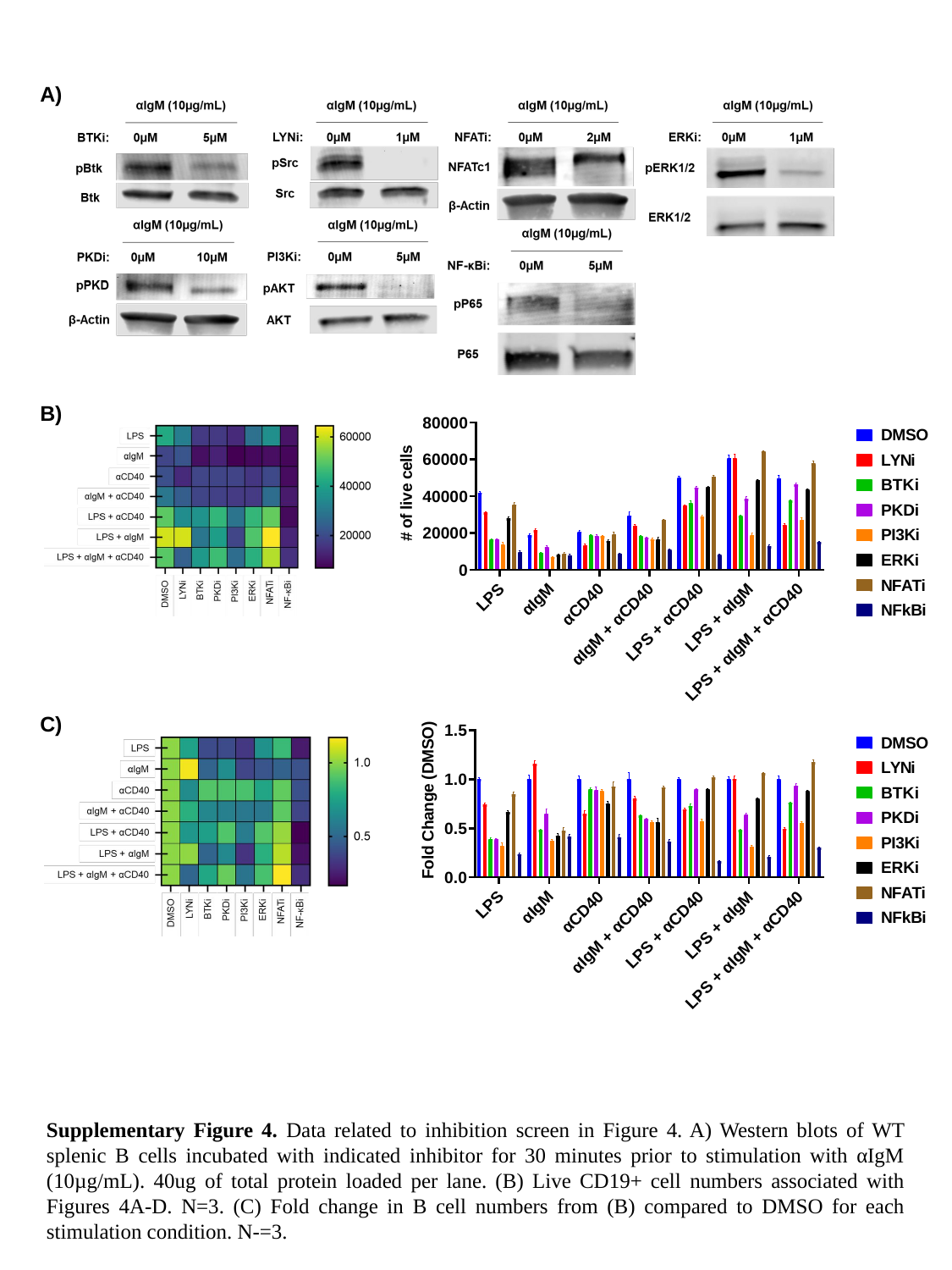

A)
B)
C)
Supplementary Figure 4. Data related to inhibition screen in Figure 4. A) Western blots of WT splenic B cells incubated with indicated inhibitor for 30 minutes prior to stimulation with αIgM (10µg/mL). 40ug of total protein loaded per lane. (B) Live CD19+ cell numbers associated with Figures 4A-D. N=3. (C) Fold change in B cell numbers from (B) compared to DMSO for each stimulation condition. N-=3.

## Slide 5
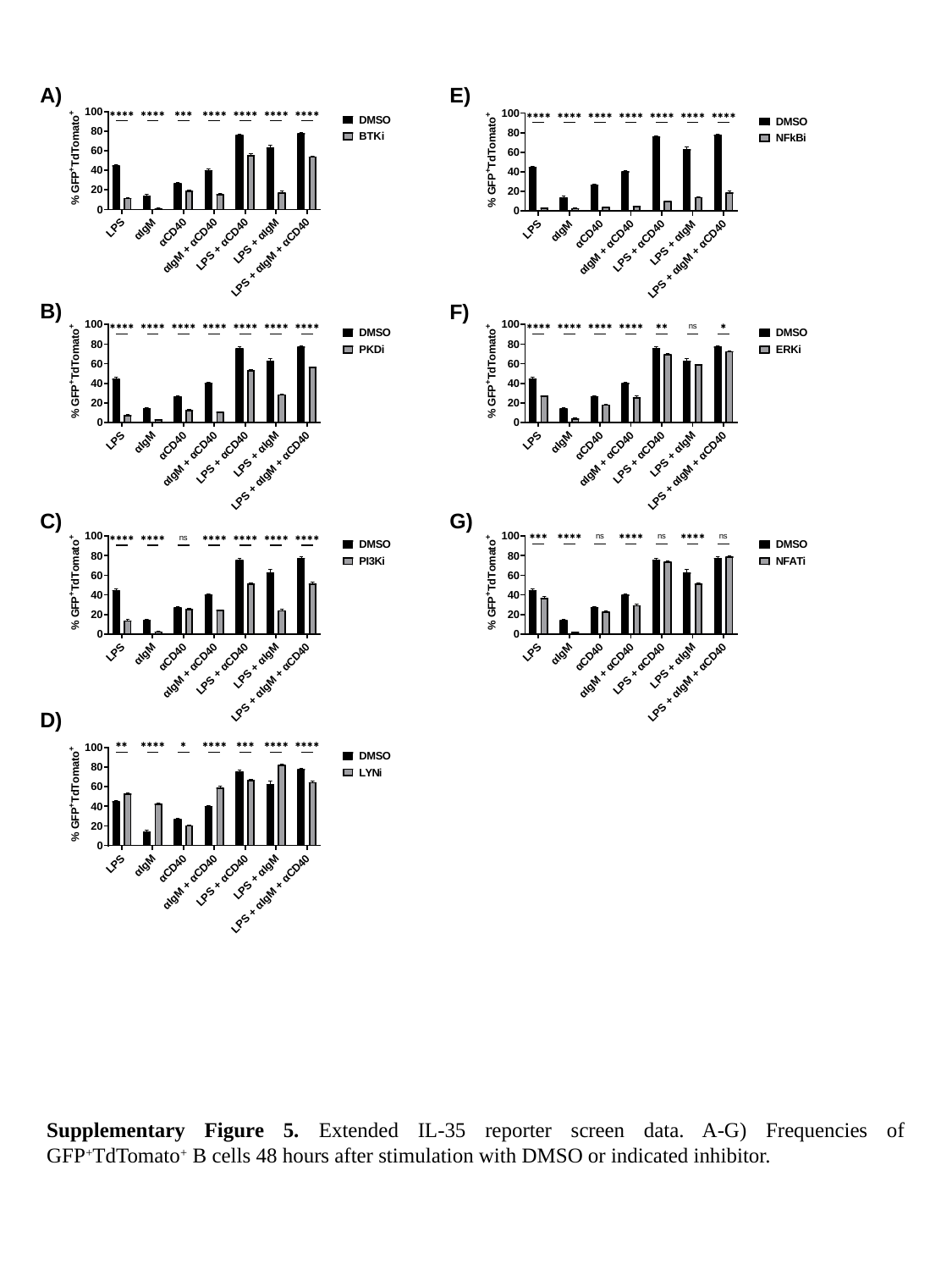

A)
E)
B)
F)
C)
G)
D)
Supplementary Figure 5. Extended IL-35 reporter screen data. A-G) Frequencies of GFP+TdTomato+ B cells 48 hours after stimulation with DMSO or indicated inhibitor.

## Slide 6
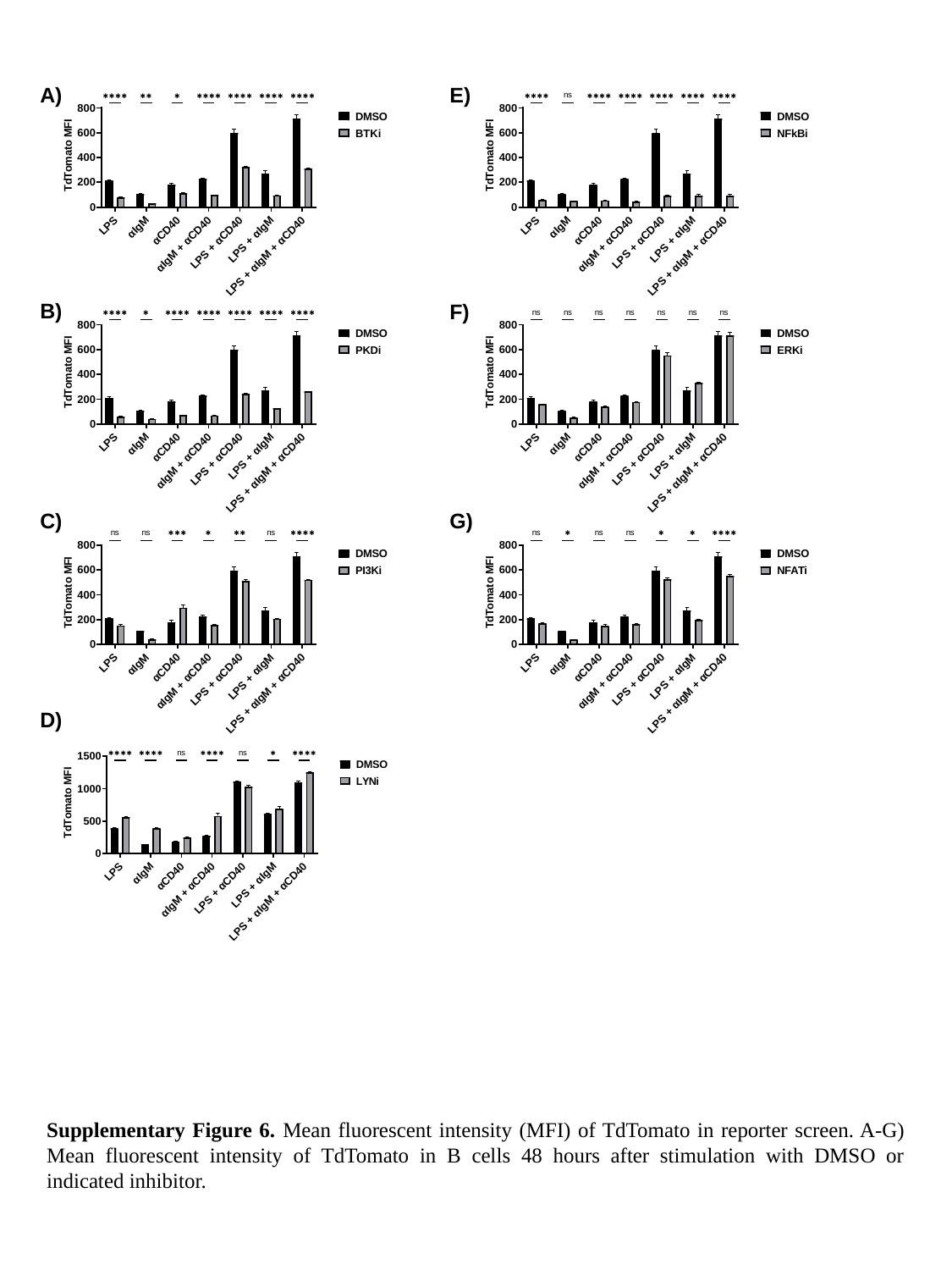

A)
E)
B)
F)
C)
G)
D)
Supplementary Figure 6. Mean fluorescent intensity (MFI) of TdTomato in reporter screen. A-G) Mean fluorescent intensity of TdTomato in B cells 48 hours after stimulation with DMSO or indicated inhibitor.

## Slide 7
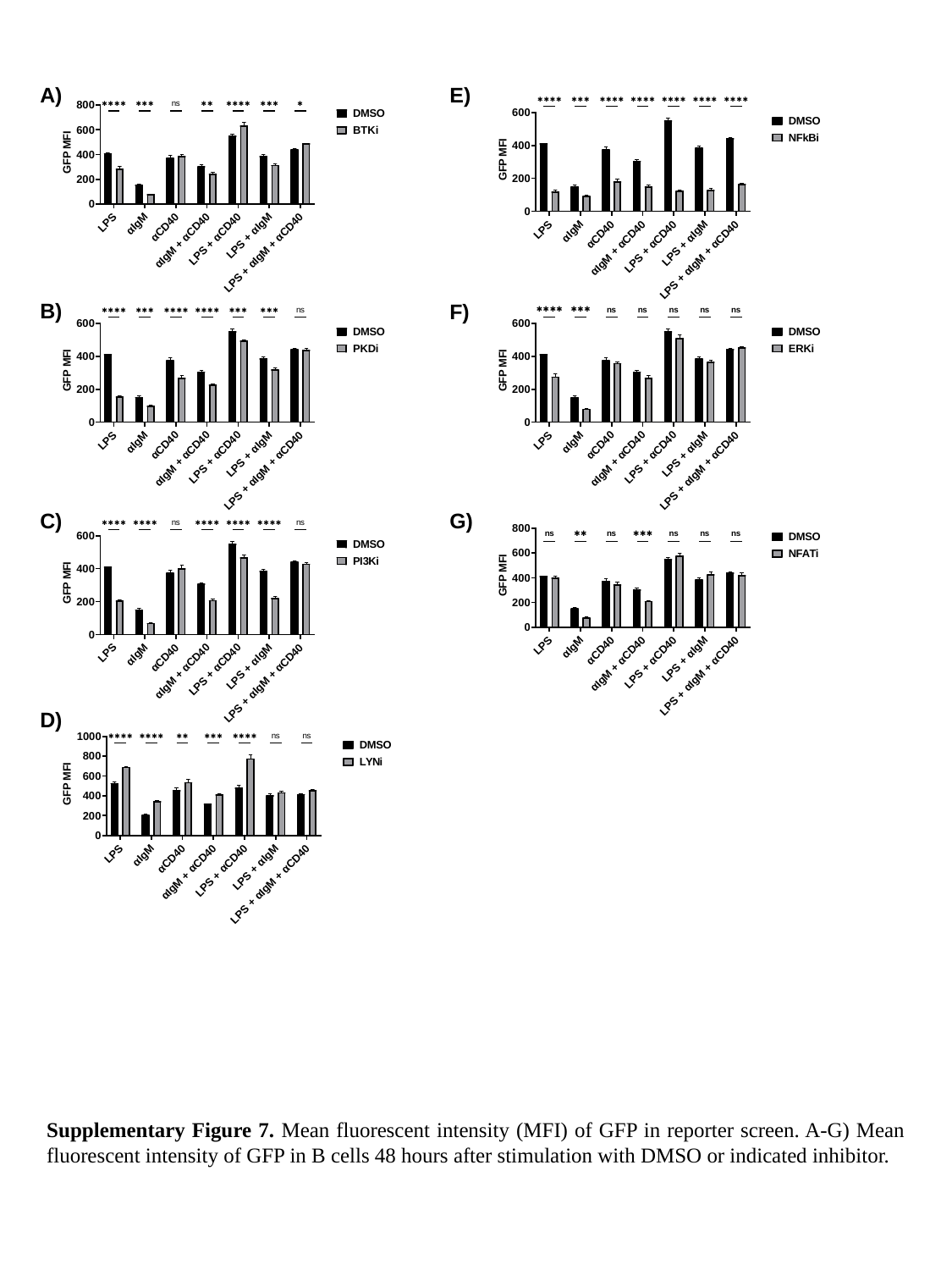

A)
E)
B)
F)
C)
G)
D)
Supplementary Figure 7. Mean fluorescent intensity (MFI) of GFP in reporter screen. A-G) Mean fluorescent intensity of GFP in B cells 48 hours after stimulation with DMSO or indicated inhibitor.

## Slide 8
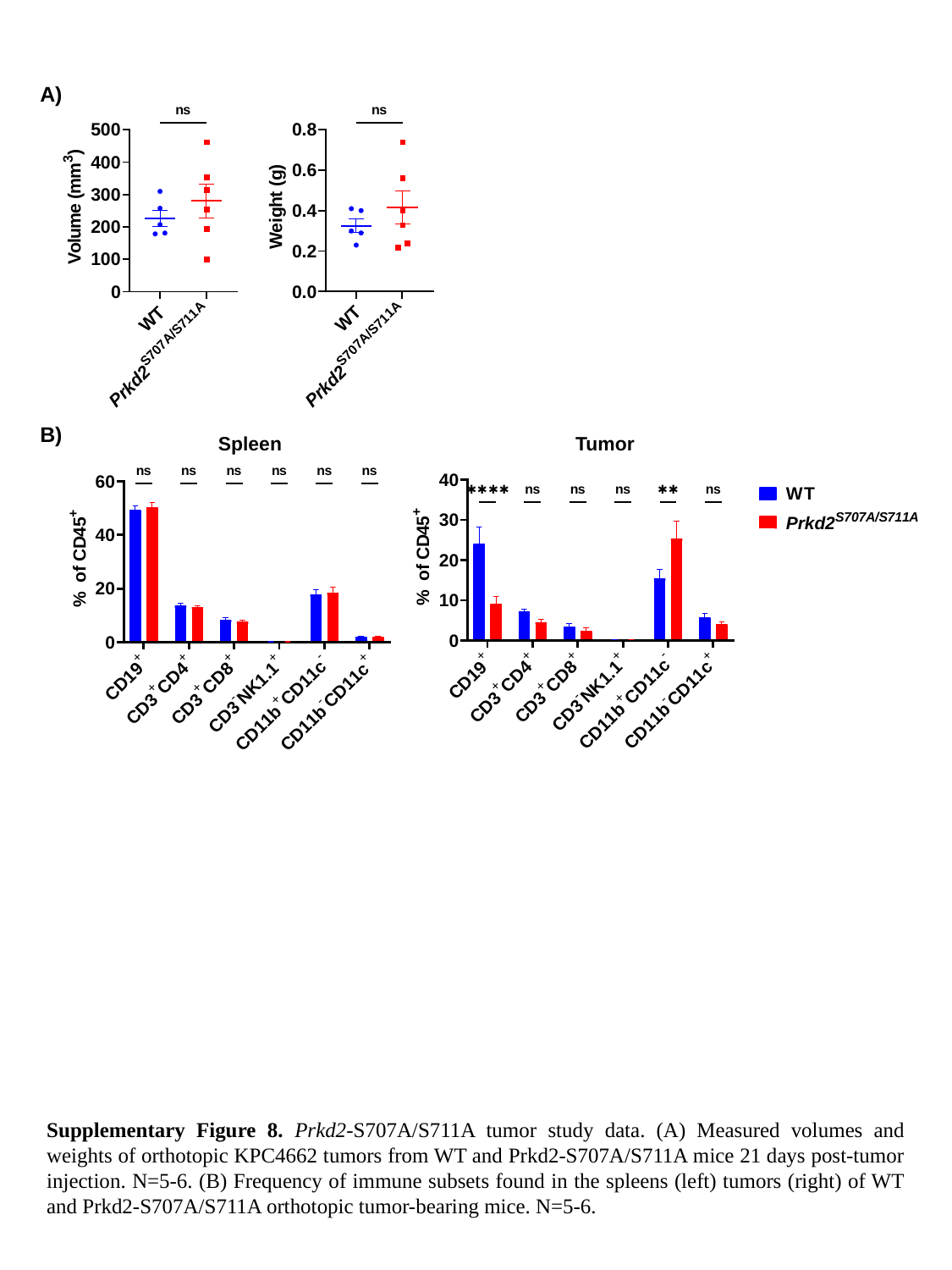

A)
B)
Spleen
Tumor
Supplementary Figure 8. Prkd2-S707A/S711A tumor study data. (A) Measured volumes and weights of orthotopic KPC4662 tumors from WT and Prkd2-S707A/S711A mice 21 days post-tumor injection. N=5-6. (B) Frequency of immune subsets found in the spleens (left) tumors (right) of WT and Prkd2-S707A/S711A orthotopic tumor-bearing mice. N=5-6.

## Slide 9
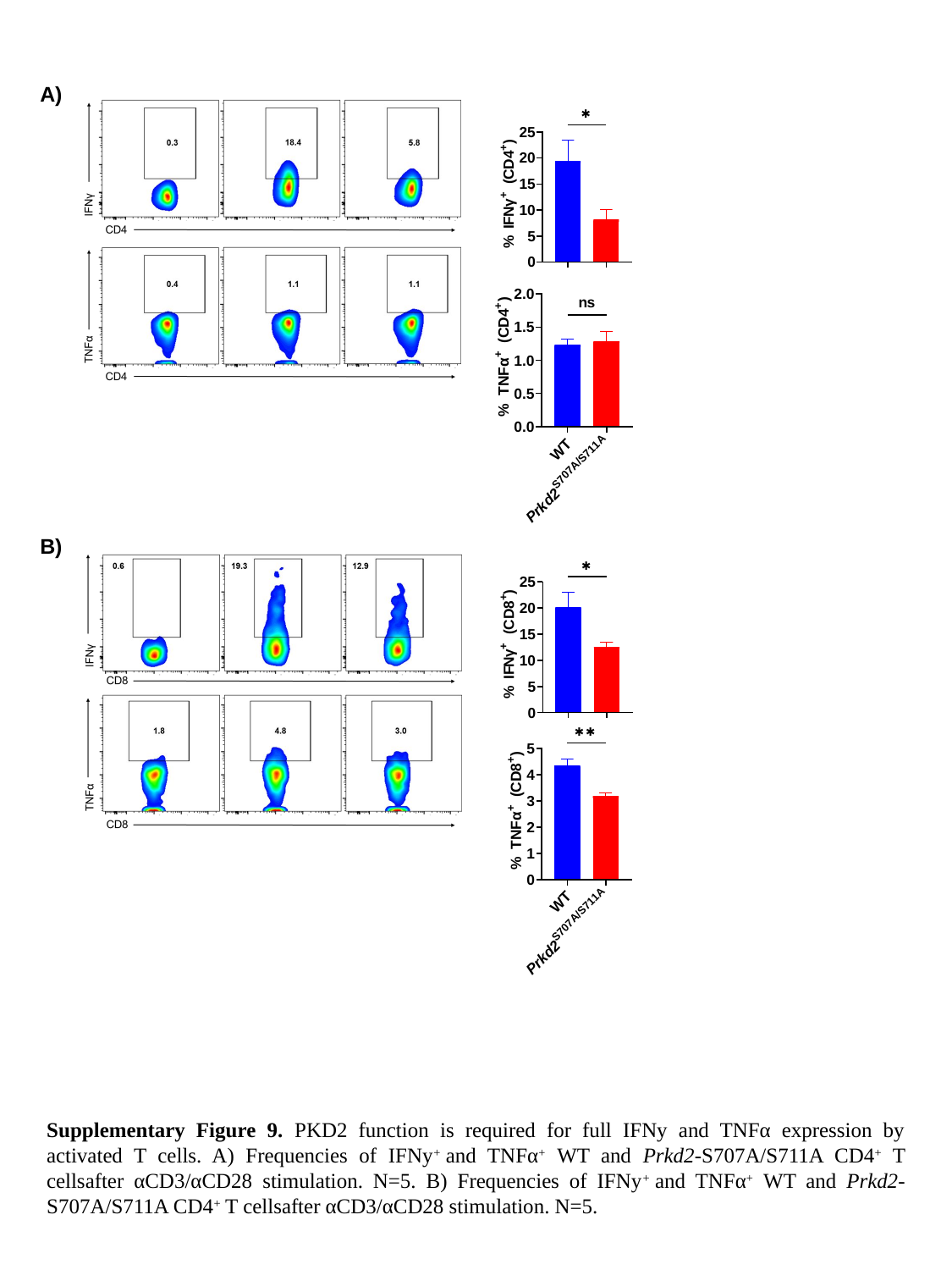

A)
B)
Supplementary Figure 9. PKD2 function is required for full IFNy and TNFα expression by activated T cells. A) Frequencies of IFNy+ and TNFα+ WT and Prkd2-S707A/S711A CD4+ T cellsafter αCD3/αCD28 stimulation. N=5. B) Frequencies of IFNy+ and TNFα+ WT and Prkd2-S707A/S711A CD4+ T cellsafter αCD3/αCD28 stimulation. N=5.

## Slide 10
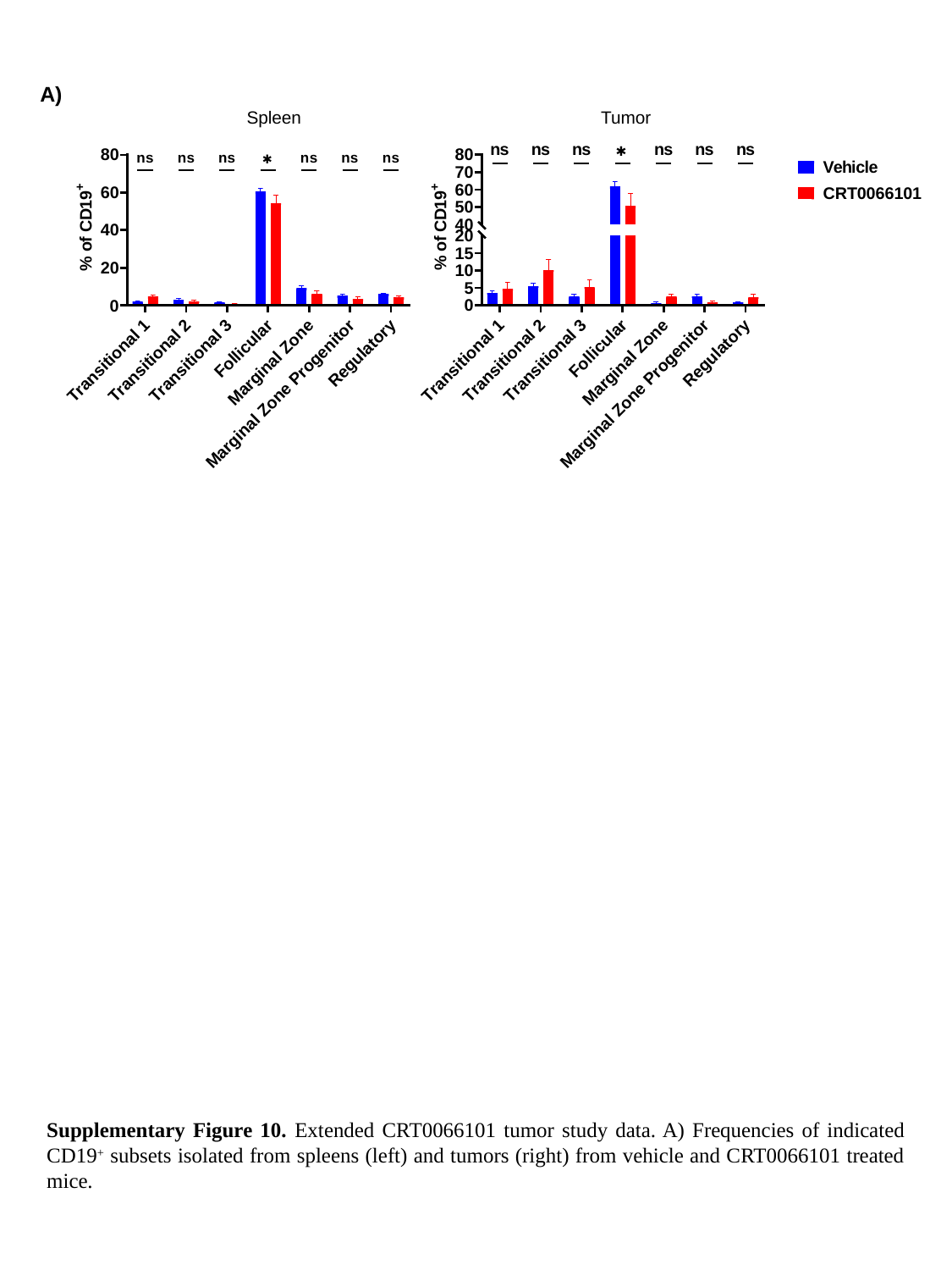

A)
Spleen
Tumor
Supplementary Figure 10. Extended CRT0066101 tumor study data. A) Frequencies of indicated CD19+ subsets isolated from spleens (left) and tumors (right) from vehicle and CRT0066101 treated mice.

## Slide 11
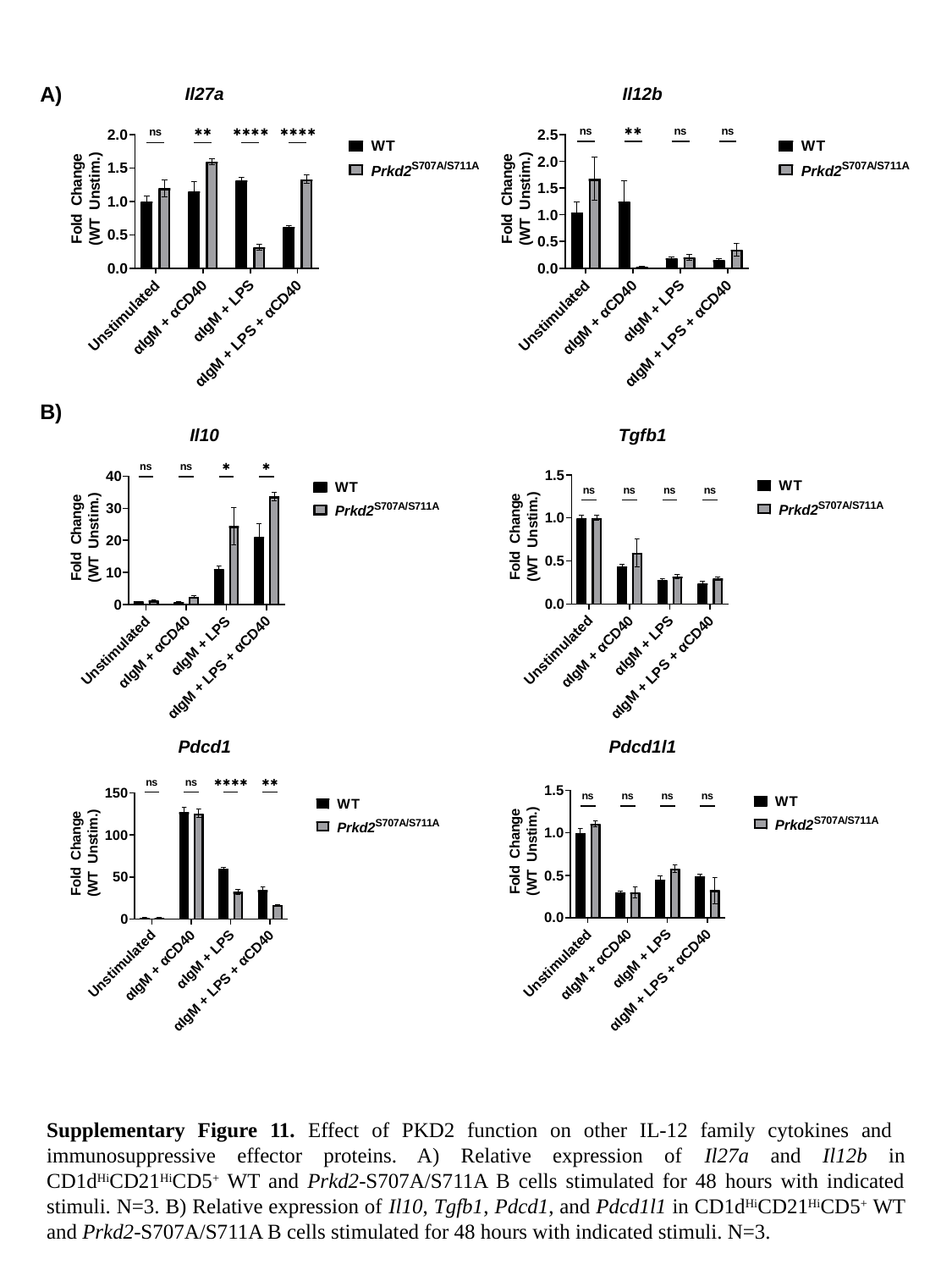

A)
Il27a
Il12b
B)
Il10
Tgfb1
Pdcd1
Pdcd1l1
Supplementary Figure 11. Effect of PKD2 function on other IL-12 family cytokines and immunosuppressive effector proteins. A) Relative expression of Il27a and Il12b in CD1dHiCD21HiCD5+ WT and Prkd2-S707A/S711A B cells stimulated for 48 hours with indicated stimuli. N=3. B) Relative expression of Il10, Tgfb1, Pdcd1, and Pdcd1l1 in CD1dHiCD21HiCD5+ WT and Prkd2-S707A/S711A B cells stimulated for 48 hours with indicated stimuli. N=3.
